# Mechanical instability generated by Myosin 19 contributes to mitochondria cristae architecture and OXPHOS

**DOI:** 10.1038/s41467-022-30431-3

**Published:** 2022-05-13

**Authors:** Peng Shi, Xiaoyu Ren, Jie Meng, Chenlu Kang, Yihe Wu, Yingxue Rong, Shujuan Zhao, Zhaodi Jiang, Ling Liang, Wanzhong He, Yuxin Yin, Xiangdong Li, Yong Liu, Xiaoshuai Huang, Yujie Sun, Bo Li, Congying Wu

**Affiliations:** 1 Institute of Systems Biomedicine, School of Basic Medical Sciences, Peking University Health Science Center, Beijing, 100191 China; 2 Institute of Biomechanics and Medical Engineering, Applied Mechanics Laboratory, Department of Engineering Mechanics, Tsinghua University, Beijing, 100084 China; 3 State Key Laboratory of Membrane Biology, Biomedical Pioneer Innovation Center (BIOPIC), School of Life Sciences, Peking University, Beijing, 100871 China; 4 National Institute of Biological Sciences, Tsinghua Institute of Multidisciplinary Biomedical Research, Tsinghua University, Beijing, 102206 China; 5 Department of Biochemistry and Biophysics, School of Basic Medical Sciences, Peking University Health Science Center, Beijing, 100191 China; 6 Group of Cell Motility and Muscle Contraction, State Key Laboratory of Integrated Management of Pest Insects and Rodents, Institute of Zoology, Chinese Academy of Sciences, Beijing, 100101 China; 7 Jiangsu Center for the Collaboration and Innovation of Cancer Biotherapy, Cancer Institute, Xuzhou Medical University, Xuzhou, Jiangsu China; 8 Biomedical Engineering Department, Peking University, Beijing, 100191 China; 9 International Cancer Institute, Peking University, Beijing, 100191 China

**Keywords:** Myosin, Energy metabolism, Mitochondria

## Abstract

The folded mitochondria inner membrane-cristae is the structural foundation for oxidative phosphorylation (OXPHOS) and energy production. By mechanically simulating mitochondria morphogenesis, we speculate that efficient sculpting of the cristae is organelle non-autonomous. It has long been inferred that folding requires buckling in living systems. However, the tethering force for cristae formation and regulation has not been identified. Combining electron tomography, proteomics strategies, super resolution live cell imaging and mathematical modeling, we reveal that the mitochondria localized actin motor-myosin 19 (Myo19) is critical for maintaining cristae structure, by associating with the SAM-MICOS super complex. We discover that depletion of Myo19 or disruption of its motor activity leads to altered mitochondria membrane potential and decreased OXPHOS. We propose that Myo19 may act as a mechanical tether for effective ridging of the mitochondria cristae, thus sustaining the energy homeostasis essential for various cellular functions.

## Abstract (web summary)

The structure of the mitochondrial inner membrane, or cristae, is important for functional oxidative phosphorylation and energy production. Here, the authors show that loss of myosin 19 impairs cristae structure as well as energy production, connecting motor activity to membrane potential.

## Introduction

Mitochondria are cellular powerhouse. Since Palade’s first electron micrographs of mitochondria in the 1950s showing a folded inner membrane encapsulated by an outer membrane^1^, the ultrastructure of mitochondria has fascinated generations of cell biologists and biophysicists for its intricate and highly organized topology in different tissues and cell types. The unique folded inner mitochondria membrane-cristae are the hubs for ATP generation^2,3^ with the respiratory chain complexes predominantly found along the length of the crista membrane and F1F0-ATPase complexes located towards the apex^4–6^. Accordingly, disruption of cristae leads to mitochondria dysfunction, resulting in reduced ATP synthesis and altered cellular metabolism^7,8^. Erenow, the SAM-MICOS (mitochondria contact site and cristae organizing system) super complex^9,10^ has been shown in different cell types to maintain mitochondria inner membrane architecture^11–13^. MICOS is a multi-subunit protein complex resided at cristae junctions (CJs)^14^ and makes contacts with the SAM (sorting and assembly machinery) complex embedded in the outer membrane^15^, forming the mitochondria intermembrane space bridging super complex^16^. Depletion of the critical components of the SAM-MICOS super complex is accompanied by downregulation of mitochondria membrane potential and ATP concentration^17–19^. Moreover, separation of SAM and MICOS, even in the presence of intact individual complexes, also leads to reduced CJ numbers and remarkable ultrastructural changes ranging from striking geometric angles to concentric onion-like circles^20^, indicating that the integrity of this super complex is required to sustain cristae architecture. Morphological regulation through mechanical force has been well studied, as in the formation of clathrin coated pits^21^ and reshaping of the nucleus during confined migration^22^. While mechanical instabilities have been generally identified in living systems to trigger wrinkling and folding during morphogenesis^23–26^, little is known about their potential contributions in the establishment of the highly convoluted mitochondria inner membrane. Thus, it is intriguing to pinpoint the role of molecular tethers in mitochondria inner membrane sculpturing.

Multiple actin motors can exert tethering force^27–30^. Among the more than 40 myosins identified so far, Myosin 19 (Myo19) is the only one that localizes onto mitochondria^31^. In vitro studies have revealed that Myo19 is a plus-end-directed high-duty ratio motor. Although it shares 30–40% motor domain sequence homology with the classical trafficking motor Myo5a^32,33^, the actin gliding velocity of Myo19 is drastically lower than that of Myo5a^32^, arguing against it being an efficient transporter. Interestingly, overexpressing Myo19 reveals an actin-dependent gain-of-function where the majority of mitochondria display a tadpole-like shape^31^, in resemblance to winning on one side in a tug-of-war and suggestive of unbiased tensile force. By simulating mitochondria ultrastructural morphogenesis in a thermodynamic model, we postulate that efficient surface folding of the cristae requires cytoplasmic intervention to initiate localized buckling. Using electron tomography, proteomics strategies and super resolution Hessian Structured illumination microscopy (Hessian-SIM) live cell imaging, we reveal that Myo19 is critical for maintaining cristae structure, by associating with the SAM-MICOS super complex. We further discover that depletion of Myo19 or disruption of its motor activity leads to aberrant mitochondria membrane potential and decreased OXPHOS. Our results suggest a novel role of mechanical tethering by Myo19 in effective cristae folding, thus stabilizing the structural foundation for energy homeostasis in cells.

## Results

### Mechanical modeling of mitochondria inner membrane reveals the role of CJ constrains in cristae morphogenesis

**Fig. 1:**
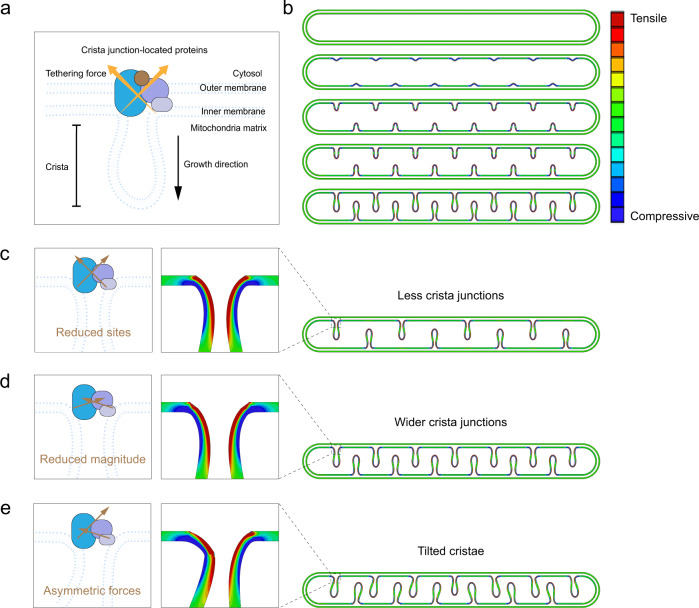
Mechanical modeling of mitochondria inner membrane reveals the role of constrains at cristae junctions in cristae morphogenesis. **a** Schematic diagram showing cristae formation. The structure of cristae, cytosol, outer membrane, inner membrane, mitochondria matrix and cristae junctions (CJs)-located proteins are illustrated. CJ-located proteins exerted tethering force on CJs. **b** Mechanical modelling of cristae growth under normal conditions. CJ-located proteins exerted tethering force on CJs and initiated inner membrane ridges. Color bar: membrane stress. **c** Lack of key CJ-located protein resulted in reduced sites of tethering force and less CJs. Left: schematic diagram showing CJ with tethering site. Right: mechanical modelling of decreased CJs’ number. Color indicates membrane stress. **d** Reduced tethering force induced wider CJs. Left: schematic diagram showing CJ with reduced tethering forces. Right: mechanical modelling of wider CJs. Color indicates membrane stress. **e** Asymmetric tethering force induced tilted crista. Left: schematic diagram showing CJ with symmetric tethering force. Right: mechanical modelling of cristae with different angles. Color indicates membrane stress.

From a mechanical perspective, structures that control the tethering strength at both ends of the CJs are required to connect the inner and outer mitochondrial membranes, as shown in Fig. 1a. To verify this, we established a two-dimensional mechanical model based on the finite element method (Abaqus 6.14), where a layered structure consisting of two thin layers, representing the inner and outer mitochondrial membranes, was considered (Fig. 1b). Both of these membranes were connected through protein complex, such as SAM-MICOS complex at CJs, where the outer membrane was assumed to provide displacement constraints to the inner membrane. Such displacement constraints equivalently gave rise to tethering forces that inhibit disconnecting and sliding of the inner membrane. Denser CJ constrains will lead to larger tethering force and, thus, induce a relatively stronger displacement constraint for the inner membrane. We introduced thermal expansion into the inner layer to mimic the synthesis of the inner mitochondrial membrane^34^. This strategy has been extensively employed to simulate tissue growth^35,36^. Our model well captured the formation and evolution of mitochondrial cristae (Fig. 1b and Supplementary Movie. 1), and showed that more CJ constrains were required to generate more inner ridges and vice versa (Fig. 1c). When the density of CJ constrains was too low, which caused low tethering forces, there were rare sites to produce inner cristae. In this scenario, the growth of the inner mitochondrial membrane will even render abnormal mitochondrial morphology (Supplementary Fig. 1a). In addition, the left–right symmetrical sliding between the inner and outer membranes may increase the width of the CJs (Fig. 1d). Moreover, asymmetrical sliding resulted in tilted cristae (Fig. 1e). These simulation results reveal the mechanical role of CJ proteins in generating and maintaining stable mitochondrial cristae.

### Myo19 is required for proper structural organization of mitochondria cristae

**Fig. 2:**
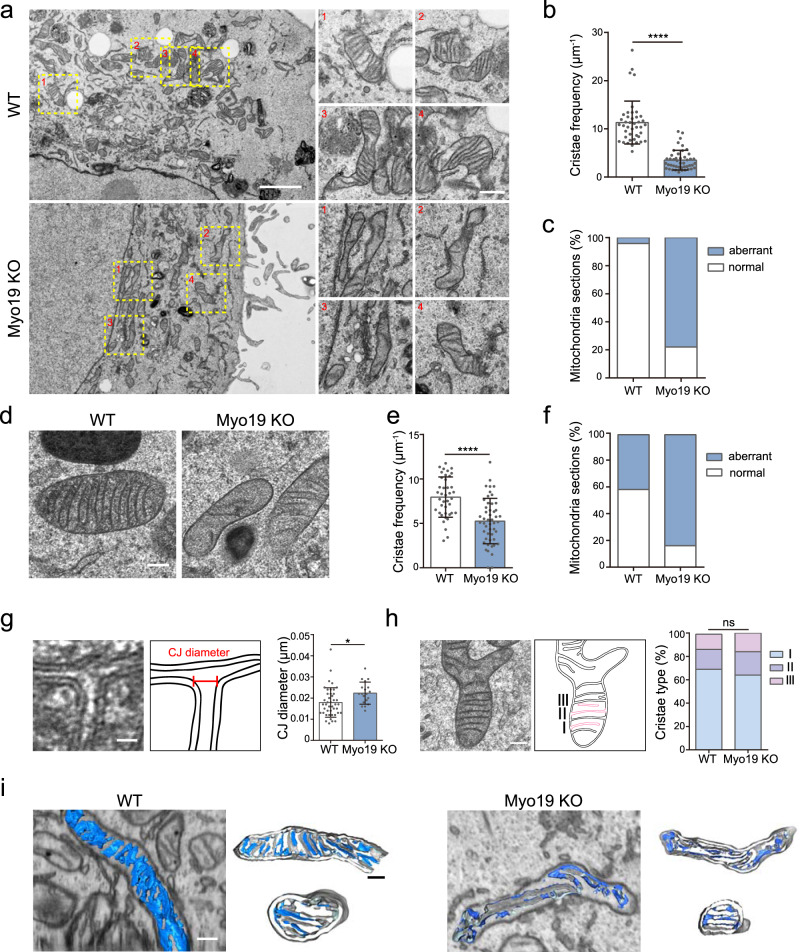
Myo19 is required for proper structural organization of mitochondria cristae. **a** Representative electron microscope (EM) images of MDA-MB-231 wild type (WT) and Myo19 knockout (Myo19 KO) cells by chemical-fixation. The yellow dashed boxes are zoomed in on the right. Scale bar: 2 μm (500 nm in magnification). **b** Quantification of the overall cristae frequency on EM images by chemical-fixation. The cristae frequency was defined as cristae numbers divided by mitochondria length. Data are shown as mean ± SD. *N*_WT_ = 42, *N*_Myo19 KO_ = 45 mitochondria. ****P < 0.0001. Significance (*P*-value) was evaluated by two-sided *t*-test. Source data are provided as a Source Data file. **c** Quantification of the percentage of cristae morphology (Mitochondrial sections) on EM images by chemical-fixation. Aberrant mitochondria included mitochondrial with reduced cristae frequency, disordered arrangement, or cristae devoid of CJs. *N*_WT_ = 332, *N*_Myo19 KO_ = 221 mitochondria. Source data are provided as a Source Data file. **d** Representative EM images of WT and Myo19 KO cells by cryo-fixation, as indicated. Scale bar: 200 nm. **e** Quantification of the overall cristae frequency on EM images by cryo-fixation. Data are shown as mean ± SD. *N*_WT_ = 40, *N*_Myo19 KO_ = 49 mitochondria. ****P < 0.0001. Significance (*P*-value) was evaluated by two-sided *t*-test. Source data are provided as a Source Data file. **f** Quantification of the percentage of cristae morphology on EM images by cryo-fixation. *N*_WT_ = 48, *N*_Myo19 KO_ = 54 mitochondria. Source data are provided as a Source Data file. **g** Left: representative EM image of CJ in WT cells by cryo-fixation. The outlines of cristae are delineated with black lines and CJ diameter is marked in red. Scale bar: 20 nm. Right: quantification of CJ diameter on EM images. Data are shown as mean ± SD. *N*_WT_ = 43, *N*_Myo19 KO_ = 19 CJs. *P = 0.0168. Significance (*P*-value) was evaluated by two-sided *t*-test. Source data are provided as a Source Data file. **h** Left: representative EM images of different types of mitochondria cristae morphology by cryo-fixation. “I” represents crista with one CJ joined to the inner boundary membrane. “II” represents crista with two CJs. “III” represents crista with no CJ. The outlines of mitochondria are delineated with black lines and three types of the representative cristae are marked in red. Scale bar: 200 nm. Right: quantification of the percentage of three types of cristae morphology. *N*_WT_ = 275, *N*_Myo19 KO_ =  202 CJs, “ns” indicates no significance. Significance (*P*-value) was evaluated by Chi-square test. Source data are provided as a Source Data file. **i** Representative images of WT and Myo19 KO mitochondria reconstructed from focused ion beam milling-scanning electron microscopy (FIB-SEM) images with different perspectives, as indicated. The outer membrane and inner boundary membrane are displayed in grey. The cristae are displayed in blue. Scale bar: 200 nm.

We then asked whether the unique mitochondria-localized actin motor-Myo19 could contribute to mitochondria cristae integrity. We generated Myo19 deficient cells (Supplementary Fig. 2a, b, Myo19 knockout cells) and detected severely disorganized mitochondria inner membrane, as revealed by thin section electron microscope (EM) with chemical- (Fig. 2a, Supplementary Fig. 2e, f) and cryo- (Fig. 2d) fixations. Notably, Myo19 depletion dramatically reduced cristae frequency (Fig. 2b, e, Supplementary Fig. 2c, d, g, Myo19 knockdown cells) while increased mitochondria morphological abnormality (Fig. 2c, f). In Myo19 knockout (KO) cells, we observed enlarged CJ diameter (Fig. 2g), as well as increased cristae that are tilted (with aberrant geometric angles in non-parallel arrangement which lead to decreased average CJ angle) (Supplementary Fig. 2h). We further classified each crista into three types: type I (crista with one CJ joined to the inner boundary membrane), type II (crista with two CJs) and type III (crista with no CJ)^37^. Most mitochondria cristae of MBA-MD-231 cells belonged to type I, and only few are type II or type III (Fig. 2h). Depletion of Myo19 did not alter the cristae distribution in the three categories (Fig. 2h), indicating that Myo19 was dispensable for cristae to fuse to or detach from the inner boundary membrane.

In order to gain insight into the 3D cristae organizational differences upon loss of Myo19, we performed focused ion beam milling combined with scanning electron microscopy (FIB-SEM). Consistent with previous reports^38^, wild type (WT) cells harbored multi-lamellar cristae, oriented perpendicular to the longitudinal axis of the mitochondria tubule and arranged parallel to each other in close proximity. We also observed occasional twisted cristae with CJ angels between 45° and 90°in WT cells (Fig. 2i). As expected from the thin section EM data, we found sparser CJs in the mitochondria tomograms in Myo19 KO cells and the ones detected were often with tilted inner membrane lamella (Fig. 2i). We also detected occasional multilayered, onion-like cristae (Supplementary Fig. 2i). Together, these results suggest that Myo19 plays an important role in regulating mitochondria cristae ultrastructure.

### Myo19 interacts with the SAM-MICOS super complex and resides in proximity to the mitochondria crista junctions

**Fig. 3:**
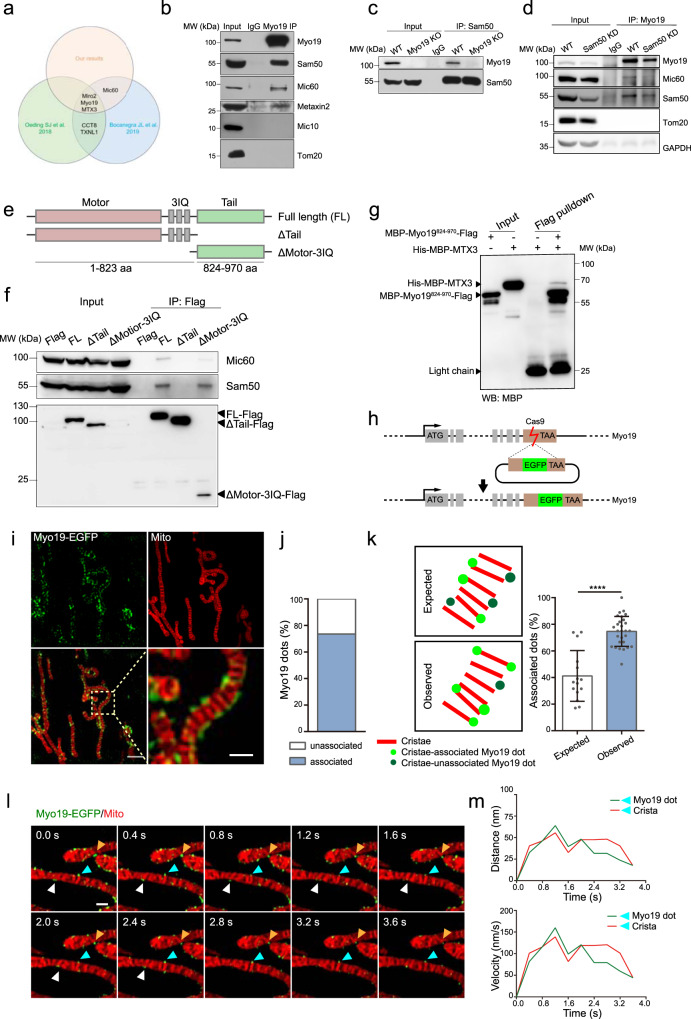
Myo19 interacts with the SAM-MICOS super complex and spatially resides in proximity to the mitochondria crista junctions. **a** Venn diagram of our IP-MS results and the two previously published proteomics studies using proximity labelling-based methods. **b** Co-immunoprecipitation of Myo19 with endogenous Sam50, Mic60, Metaxin2 and Mic10 in MDA-MB-231 WT cells. The immunoprecipitates are blotted as indicated. Source data are provided as a Source Data file. **c** Co-immunoprecipitation of Sam50 with endogenous Myo19 in WT and Myo19 KO cells. The immunoprecipitates are blotted as indicated. Source data are provided as a Source Data file. **d** Co-immunoprecipitation of Myo19 with endogenous Sam50 and Mic60 in WT and Sam50 knockdown (Sam50 KD) cells. GAPDH was used as loading control. The immunoprecipitates are blotted as indicated. Source data are provided as a Source Data file. **e** Schematic diagram showing the Myo19 truncations: full length (FL) Myo19 (1-970 aa), ΔTail (1-823 aa), ΔMotor-3IQ (824-970 aa). Flag is tagged at the C terminus of each truncation. **f** Co-immunoprecipitation of Myo19 truncations with endogenous Sam50 and Mic60. The immunoprecipitates are blotted as indicated. The Myo19 truncations were expressed in HEK293T cells. Source data are provided as a Source Data file. **g** In vitro pull-down assay of the purified MBP-Myo19^824-970^-Flag and His-MBP-MTX3. Source data are provided as a Source Data file. **h** Schematic diagram showing the principle of Myo19-EGFP knock-in cell line construction. **i** Representative images of MDA-MB-231 Myo19-EGFP knock-in cells stained with 200 nM MitoTracker^TM^ Red CMXRos (Mito) for 15 min. The yellow dashed box is zoomed in on the right. The images were captured by Hessian-SIM super resolution microscope. Scale bar: 2 μm (500 nm in magnification) **j** Quantification of the percentage of cristae-associated and -unassociated Myo19 dots. *N*_Total_ = 443. Source data are provided as a Source Data file. **k** Left: schematic diagram showing cristae, cristae-associated and -unassociated Myo19 dots. Right: quantification of expected (random distribution in theory) and observed cristae-associated Myo19 dots proportion. To estimate the theoretical distribution rate of randomly distributed dots on cristae (expected value), we used ImageJ and set threshold to determine boundaries of MitoTracker^TM^ Red CMXRos stained-cristae, and used line scan to quantify the ratios of pixels with positive fluorescence intensity to overall pixels. Data are shown as mean ± SD. *N*_expected_ = 15, *N*_observed_ = 28 dots. ****P < 0.0001. Significance (*P*-value) was evaluated by two-sided *t*-test. Source data are provided as a Source Data file. **l** Examples of temporal correlation between Myo19 dots and cristae. Myo19-EGFP knock-in cells were stained with 200 nM MitoTracker^TM^ Red CMXRos (Mito) for 20 min, and were tracked and imaged by time-lapse Hessian-SIM. Arrows of different colors indicate different cristae-associated Myo19 dots. Scale bar: 500 nm. **m** Quantification of the trajectory (Top) and velocity (Bottom) of Myo19 dot and crista indicated by cyan arrows in **j**. Source data are provided as a Source Data file.

To gain molecular insight into the role of Myo19 in regulating cristae architecture, we performed immunoprecipitation coupled to mass spectrometry (IP-MS) to search for Myo19 binding partners. By comparing our IP-MS results with the two previously published proteomics studies using proximity labelling-based methods^39,40^, we detected three common elements—Myo19, Miro2 and Metaxin3 (MTX3) in all of the three studies (Fig. 3a). Interestingly, many of the members in the SAM-MICOS super complex including Sam50 and Mic60, the essential components of the SAM and MICOS respectively, were among the major interacting proteins (Supplementary Fig. 3a). We subsequently confirmed these bindings by reciprocal co-immunoprecipitation (Co-IP) (Fig. 3b, c), and found that Myo19 level and its binding with Mic60 significantly decreased upon Sam50 knockdown (Fig. 3d) while Myo19 could still interact with Sam50 upon Mic60 depletion (Supplementary Fig. 3b). The mammalian SAM complex locates on the outer mitochondrial membrane and consists of Sam50, Metaxin1 (MTX1, known as Sam37 in yeast), Metaxin2 (MTX2, known as Sam35 in yeast) and Metaxin3^41,42^. Proximity ligation assay (PLA) further demonstrated that the Myo19-Sam50 interaction was specifically resided on mitochondria (Supplementary Fig. 3c). Myo19 contains three major domains: the N terminal motor domain and the C terminal tail domain linked by a neck region containing three tandem IQ motifs^31,32^. By evaluating the capacity of Myo19 truncations (Fig. 3e) to interact with Sam50, we identified that the Myo19 tail domain was required for Sam50 association (Fig. 3f). Moreover, we asked whether Myo19 could bind SAM complex directly. With purified proteins, we found that the tail domain of Myo19 (Myo19^824-970^) could be pulled down directly by Metaxin3 but not Metaxin2 nor Sam50 fragments (Fig. 3g and Supplementary Fig. 3d–f), indicating that Myo19 might directly interact with SAM/MICOS complex by binding to Metaxin3.

Previous studies revealed Miro1/2 as Myo19 interacting partners and that Miro1/2 could stabilize Myo19 onto mitochondria^39,40,43,44^. These studies promoted us to explore the dependency of Myo19 mediated cristae regulation on Miro1/2. By generating Miro1/2 double knockout cells, we found that exogenously expressed full length Myo19-EGFP could still localize on mitochondria although it showed more cytoplasmic distribution (Supplementary Fig. 4a,b). Moreover, we also detected the interaction between Myo19 and Sam50 in Miro1/2 double knockout cells by BiFC (bimolecular fluorescence complementation), though the signal seemed weaker than in control cells (Supplementary Fig. 4c). These results indicated that the interaction between Myo19 and Sam50 might not necessarily require Miro1/2, but the presence of Miro1/2 might enhance this interaction.

To examine the in situ localization of Myo19, we generated a knock-in cell line where EGFP was inserted into the C terminus of the Myo19 gene locus (Fig. 3h). The knock-in efficacy was examined by immunoblotting (Supplementary Fig. 5a). Consistent with previous reports using exogenous system^31^, the endogenously expressed Myo19-EGFP exhibited specific mitochondria localization (Supplementary Fig. 5b). We then employed the Hessian-SIM super resolution microscopy^45^ and the Ultrastructure expansion microscopy (U-ExM)^46,47^ to visualize endogenous Myo19 distribution in cells. Notably, we detected discontinuous Myo19-EGFP signals along the mitochondria (Fig. 3i and Supplementary Fig. 5c), reminiscent of what has been observed with Sam50, Mic60 and Miro1/2^20,48,49^.

Of note, the majority of Myo19 spots were spatially associated with CJs, which we defined as Myo19 dots that overlapped with cristae and with overlapping area occupying more than half of its own size (Fig. 3j), and the spatial correlation was statistically higher than the expected random distribution (Fig. 3k). In support of the notion that Myo19 functionally coordinated with the SAM-MICOS super complex, we observed high spatial-temporal correlation between Myo19 and the cristae that the Myo19 signals displayed synchronized motions with approximal cristae (Fig. 3l, m). Together, these results suggest that Myo19 interacts with the SAM-MICOS super complex and spatially associates with the mitochondria crista junctions.

### Myo19 maintains mitochondria membrane potential and affects OXPHOS

**Fig. 4:**
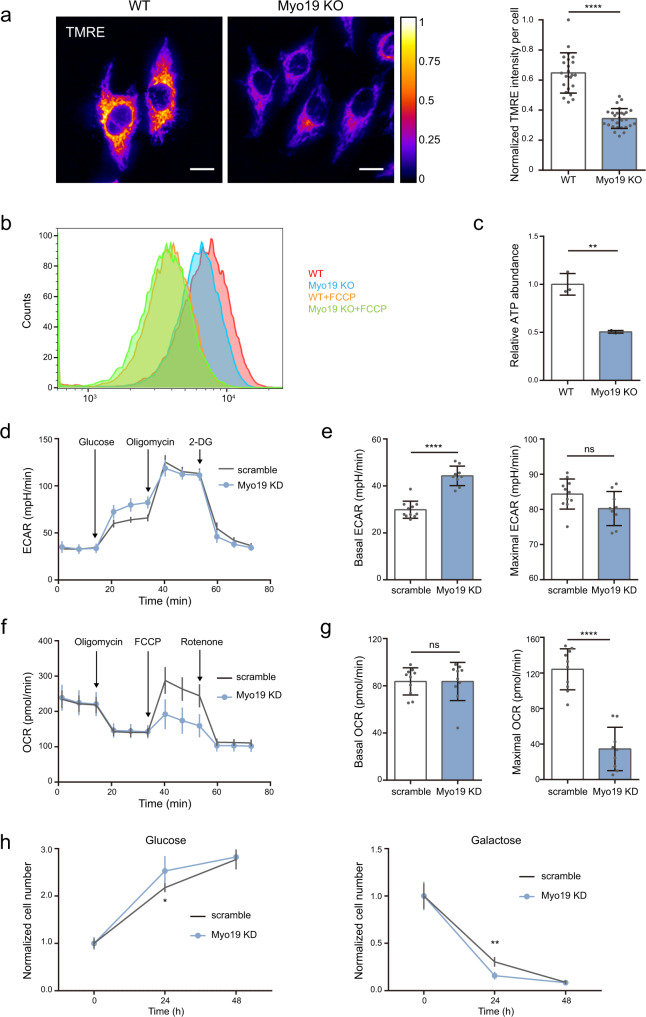
Myo19 maintains mitochondria membrane potential and affects OXPHOS. **a** Left: representative thermographs of MDA-MB-231 WT and Myo19 KO cells stained with 100 nM TMRE for 20 min. Scare bar: 20 μm. Right: quantification of TMRE fluorescence intensity per cell. Data are shown as mean ± SD. *N*_WT_ = 22, *N*_Myo19 KO_ = 28. ****P < 0.0001. Significance (*P*-value) was evaluated by two-sided *t*-test. Source data are provided as a Source Data file. **b** Flow cytometry analysis of WT and Myo19 KO cells stained with 100 nM TMRM for 20 min. 5 μM FCCP treatment for 10 min was used as positive control. Gating/sorting strategy was performed according to product manual. **c** Quantification of relative ATP abundance in WT and Myo19 KO cells. Data are shown as mean ± SD. *N*_WT_ = 3, *N*_Myo19 KO_ = 3 independent samples. **P = 0.0017. Significance (*P*-value) was evaluated by two-sided *t*-test. Source data are provided as a Source Data file. **d** The representative curves of extracellular acidification rate (ECAR) in MDA-MB-231 scramble and Myo19 knock down (Myo19 KD) cells. Glucose:4.5 g/L; Oligomycin: 1 μM; 2-DG: 50 mM. Data are shown as mean ± SD. *N*_scramble_ = 11, *N*_Myo19 KD_ = 9 independent samples. Source data are provided as a Source Data file. **e** Quantification of basal and maximal ECAR. Data are shown as mean ± SD. *N*_scramble_ = 11, *N*_Myo19 KD_ = 9 independent samples. ****P < 0.0001. ns, no significance. Significance (*P*-value) was evaluated by two-sided *t*-test. Source data are provided as a Source Data file. **f** The representative curves of oxygen consumption rate (OCR) in scramble and Myo19 KD cells. Oligomycin: 1 μM; FCCP:1 μM; Rotenone: 1 μM. Data are shown as mean ± SD. *N*_scramble_ = 10, *N*_Myo19 KD_ = 10 independent samples. Source data are provided as a Source Data file. **g** Quantification of basal and maximal OCR. ns, no significance. Data are shown as mean ± SD. *N*_scramble_ = 10, *N*_Myo19 KD_ = 10 independent samples. ****P < 0.0001. Significance (*P*-value) was evaluated by two-sided *t*-test. Source data are provided as a Source Data file. **h** Growth curves of scramble and Myo19 KD cells grown in medium supplemented with the indicated monosaccharides. Left: glucose-based medium. Data are shown as mean ± SD. *N*_scramble_ = 5, *N*_Myo19 KD_ = 5 independent samples. *P = 0.0449. Right: galactose-based medium. Data are shown as mean ± SD. *N*_scramble_ = 5, *N*_Myo19 KD_ = 5 independent samples. **P = 0.0011. Significance (*P*-value) was evaluated by two-sided *t*-test. Source data are provided as a Source Data file.

Most of the enzymes in the electron transport chain reside on the mitochondria inner membrane and are enriched on the cristae^7^. Previous study has revealed that loss of Myo19 impairs mitochondria respiratory capacity by decreasing membrane potential and downregulating oxygen consumption rate (OCR)^50^. Using the mitochondrial membrane potential probes, we also confirmed that upon Myo19 deletion, mitochondria membrane potential was significantly decreased, as revealed both from imaging quantification (Fig. 4a) and flow cytometry analysis (Fig. 4b). Decreased membrane potential oftentimes links to abnormal energy production. Indeed, Myo19 deficient cells exhibited markedly lower level of ATP, compared to WT cells (Fig. 4c). Since ATP abundance is influenced by the balance between glycolysis and OXPHOS, we then employed the Seahorse XF96 analyzer to measure the cellular oxygen consumption rate (OCR) and extracellular acidification rate (ECAR) for glucose oxidation evaluation (Fig. 4d–g). No statistically significant differences in the maximal ECAR were detected between WT and Myo19 knockdown (KD) cells (Fig. 4d, e). However, the maximal OCR dramatically downregulated in Myo19 KD cells, consistent with previous finding^50^ and in agreement with the cristae structure defects we observed in these cells (Fig. 4f, g). To better address if Myo19 depletion affected mitochondria-dependent cell growth, we assayed cell viability in galactose media, where most of the cellular ATP comes from the respiratory chain^7^. Consistent with decreased OCR measured by Seahorse, Myo19 deficient cells also exhibited reduced galactose utilization (Fig. 4h), indicating impaired OXPHOS. We further evaluated the protein levels of OXPHOS-related proteins including MT-ND1 (complex I of electron transfer chain), MT-CYB (complex III of electron transfer chain), COX-IV (complex IV of electron transfer chain) and MT-ATP8 (ATP synthase F0 subunit 8). The protein levels of MT-ATP8, MT-ND1 and COX-IV were not changed, while the MT-CYB protein level in Myo19 knockout cells decreased (Supplementary Fig. 6). Collectively, these data unveil a critical role of Myo19 in maintaining mitochondria membrane potential and OXPHOS in cells.

### The motor activity of Myo19 is indispensable in regulating mitochondria ultrastructure and function

**Fig. 5:**
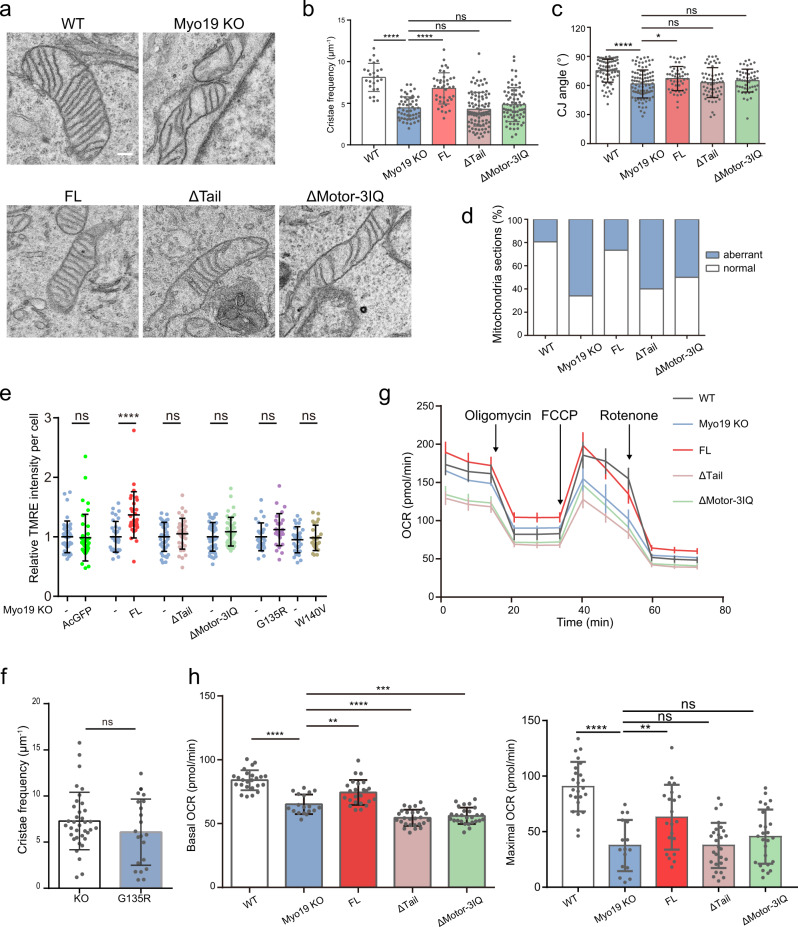
The motor activity of Myo19 is indispensable in regulating mitochondria ultrastructure and function. **a** Representative EM images of mitochondria cristae in MDA-MB-231 WT, Myo19 KO and Myo19 KO cells rescued with full length Myo19 (FL), motor truncation (ΔTail) and tail truncation (ΔMotor-3IQ). Scale bar: 200 nm. **b** Quantification of the overall cristae frequency on EM images. Data are shown as mean ± SD and analysed by One-way ANOVA analysis as well as Dunnett’s multiple comparisons test. *N*_WT_ = 25, *N*_KO_ = 53, *N*_FL_ = 41, *N*_ΔTail_ = 91, *N*_ΔMotor-3IQ_ = 71 mitochondria. ****P < 0.0001, ns, no significance. Significance (*P*-value) was evaluated by One-Way ANOVA and Multiple comparison. Source data are provided as a Source Data file. **c** Quantification of CJ angle on EM images. Data are shown as mean ± SD and analysed by One-way ANOVA analysis as well as Dunnett’s multiple comparisons test. *N*_WT_ = 75, *N*_KO_ = 97, *N*_FL_ = 49, *N*_ΔTail_ = 58, *N*_ΔMotor-3IQ_ = 51 CJs. *P = 0.0476, ****P < 0.0001. Significance (*P*-value) was evaluated by One-Way ANOVA and Multiple comparison. Source data are provided as a Source Data file. **d** Quantification of the cristae morphology on EM images. *N*_WT_ = 36, *N*_KO_ = 50, *N*_FL_ = 64, *N*_ΔTail_ = 70, *N*_ΔMotor-3IQ_ = 73 mitochondria. Source data are provided as a Source Data file. **e** Quantification of relative TMRE fluorescence intensity per cell in MDA-MB-231 Myo19 KO and Myo19 KO cells rescued with AcGFP, FL Myo19, ΔTail, ΔMotor-3IQ and Myo19^G135R^ mutants. Data are shown as mean ± SD. Group 1: *N*_Myo19 KO_ = 40, *N*_AcGFP_ = 36, ns, no significance; Group 2: *N*_Myo19 KO_ = 33, *N*_FL_ = 29, ****P < 0.0001; Group 3: *N*_Myo19 KO_ = 39, *N*_ΔTail_ = 34, ns; Group 4: *N*_Myo19 KO_ = 42. *N*_ΔMotor-3IQ_ = 43, ns; Group 5: *N*_Myo19 KO_ = 27, *N*_G135R_ = 27, ns. Significance (*P*-value) was evaluated by two-sided *t*-test. Source data are provided as a Source Data file. **f** Quantification of the cristae frequency of Myo19 KO and Myo19^G135R^ cells. Data are shown as mean ± SD. *N*_KO_ = 36, *N*_G135R_ = 21 mitochondria. Significance (*P*-value) was evaluated by two-sided *t*-test. Source data are provided as a Source Data file. **g** The representative curves of OCR of MDA-MB-231 WT, Myo19 KO and Myo19 KO cells rescued with FL Myo19, ΔTail and ΔMotor-3IQ truncations. Data are shown as mean ± SD. *N*_WT_ = 8, *N*_Myo19 KO_ = 6, *N*_FL_ = 7, *N*_ΔTail_ = 9, *N*_ΔMotor-3IQ_ = 9 independent samples. Source data are provided as a Source Data file. **h** Left: quantification of the basal OCR. *N*_WT_ = 24, *N*_KO_ = 18, *N*_FL_ = 24, *N*_ΔTail_ = 27, *N*_ΔMotor-3IQ_ = 27 independent samples. ****P < 0.0001, ***(Myo19 KO vs. FL), *P* = 0.0006, ***(Myo19 KO vs. ΔMotor+3IQ, *P* = 0.0007. Right: quantification of the maximal OCR. Data are shown as mean ± SD. *N*_WT_ = 24, *N*_KO_ = 16, *N*_FL_ = 21, *N*_ΔTail_ = 27, *N*_ΔMotor-3IQ_ = 26 independent samples. ****P < 0.0001, **P = 0.0060. Significance (*P*-value) was evaluated by One-Way ANOVA and Multiple comparison. Source data are provided as a Source Data file.

Our computational modeling suggested that a molecular tether would be beneficial for effective folding of the mitochondria cristae. The duty ratio of Myo19 does not support it being an efficient transporter but may suggest a role in providing tethering force^32,51^. These evidences prompted us to pinpoint whether Myo19 may engage its motor activity with cristae architecture. To do this, we introduced the full-length Myo19 or the Myo19 truncations into the Myo19 KO cells and evaluated their capacity in restoring cristae architecture and mitochondria function. By thin section EM (Fig. 5a), we validated that the full-length Myo19 was able to rescue cristae frequency (Fig. 5b), CJ angle (Fig. 5c) and mitochondria morphological abnormality (Fig. 5d). Intriguingly, both motor- and tail- truncations failed to restore cristae integrity (Fig. 5a–d). Consistently, these mutants were incompetent to rescue mitochondria membrane potential (Fig. 5e) or OXPHOS (Fig. 5g, h) in Myo19 KO cells, suggesting that actin-binding and mitochondria-targeting were both required for Myo19 to function in regulating cristae structure and mitochondria metabolism. Myosins use ATP to fuel dynamic binding with actin filaments, promoting their association with various cellular components^51^. To more specifically examine the role of Myo19 in serving as such a molecular tether, we then employed Myo19^G135R^ and Myo19^W140V^, the ATP binding and hydrolysis mutant that binds tightly to actin filaments^33,52–54^). With these mutants, we did not observe obvious recovery of mitochondria membrane potential (Fig. 5e). And G135R also failed to rescue the cristae frequency (Fig. 5f). Last but not least, we examined the requirement of the intact actin network for cristae structure. We found that actin depolymerization by latrunculin B (LatB) or cytochalasin D (CytoD) significantly impaired cristae density and morphology (Supplementary Fig. 7a,b). We then explored the role of branched and linear actin in cristae ultrastructural maintenance, by using an Arp2/3 inhibitor CK666 and a formin inhibitor SMIFH2^55–57^. Interestingly, destruction of linear actin but not branched actin decreased mitochondrial cristae frequency (Supplementary Fig. 7a,c). Yang and Svitkina have previously demonstrated that the mitochondria with no prominent constrictions interacted with actin filaments relatively uniformly along its length and most actin filaments in the interstitial network appeared to be linear actin^58^. Our results proposed a possible function of these interstitial linear actin filaments along the mitochondria surface in maintaining cristae structural integrity.

Previous study also demonstrated the role of Miro1/2 in maintaining cristae structure in a microtubule-based mechanism^49^, which prompted us to investigate whether mechanical force generated by the microtubule motors was required for cristae structure integrity. KIF5B has been shown to be responsible for microtubule-based anterograde transport of mitochondria and for the mitochondria network formation by mitochondrial tabulation^59^. Compared with WT cells, the mitochondria accumulated and surrounded cell nucleus in KIF5B knockout cells (Supplementary Fig. 7d), in accordance with loss of microtubule plus end trafficking of mitochondria upon kinesin perturbation^59^. However, when we used electron microscopy to visualize cristae morphology, we found that the cristae frequency was not affected by KIF5B depletion (Supplementary Fig. 7d). Furthermore, we explored the dependency of the intact microtubule network for mitochondria cristae structure. Nocodazole treatment depolymerized the microtubule network but did not significantly alter cristae frequency (Supplementary Fig. 7a,b), indicating that cristae formation may rely on actin instead of the microtubule cytoskeleton. Together, these experimental observations further support our mathematical model that mechanical force provided by the mitochondria-localized actin motor Myo19, may serve to maintain cristae structure and mitochondria function in cells.

## Discussion

Computational simulations have facilitated the mechanobiological studies ranging from molecular motors to organ morphogenesis. Previous mathematical modelling of mitochondria morphogenesis suggested tubular/lamellar crista structure could be stabilized by tensile force^24^. Here we employed a mechanical model and unveiled that the formation and evolution of mitochondrial cristae required tethering forces generated by CJ proteins. Although our simulations suggested that tethering forces were required to initiate inner membrane ridges, the precise force levels, which depend on the mechanical properties of CJ proteins and inner and outer membrane, remain unclear. In addition, actin filaments may form contractile ring to constrict mitochondria, as revealed from the recent platinum replica electron microscopy (PREM) studies^58^. Further mechanical quantification and in vitro reconstruction of actin-Myo19 and mitochondrial will shed new light on Myo19 function on mitochondria.

To satisfy the increased energy demands, increased mitochondrial biogenesis and elevated electron transport chain (ECT) efficiency are adopted for skeletal muscle cells. Two individual RNA sequencing studies detected significantly upregulated Myo19 mRNA level in young human males and thoroughbred horses after high intensity training^60–62^. Concerning our finding that Myo19 affects mitochondria metabolism, it is likely that Myo19 may contribute to skeletal muscle energy demands. In mammals, myocardial cells harbor the most abundant mitochondria with densely and parallelly packed cristae. Disorganized inner mitochondria membrane is a pathological signature for myriads of heart failure diseases. The potential implications of defected Myo19 activity in skeletal muscle and heart tissues are worth evaluating.

Metabolic switch from OXPHOS to glycolysis, known as Warburg effect, is a hallmark of cancer, supporting tumor growth and cancer stemness. Genome-wide association studies suggest that Myo19 may involve in ovarian cancer, pancreatic ductal adenocarcinoma and breast cancer but whether it may play a tumor suppressive role is unclear^62–67^. In Myo19 deleted MDA-MB-231 breast cancer cells, we detected decreased maximal OCR and a little increased basal ECAR, showing a tilted metabolic balance. The high basal glycolytic level of MDA-MB-231 cells may explain the unchanged maximal ECAR upon Myo19 loss. The decreased basal OCR in Myo19 KO cells but not Myo19 KD cells may suggest that marginal expression of Myo19 is likely sufficient for maintenance of basal OCR but not maximal OCR. Also, we did not observe significant cell proliferation advantages in Myo19 KO MDA-MB-231 cells. Thus, whether Myo19 mediated metabolic reprogramming is related to tumor stemness changes and Myo19 associated tumor occurrence awaits future investigation.

## Methods

### Cell culture and DNA transfection

MDA-MB-231 cells were from ATCC provided by Dr. Yujie Sun (Peking University) and HEK293T cells were provided by Dr. Yuxin Yin (Peking University). Cells were cultured in Dulbecco’s modified Eagle medium (LVN1001-1, Livning) supplemented with 10% fetal bovine serum (ST30-3302, PAN), 100 U/mL penicillin and 100 μg/mL streptomycin (CC004, MACGENE) at 37 °C with 5% CO_2_. For imaging, the cells were seeded on the fibronectin coated coverslips or glass bottom dishes. For transfection, cells were transfected with 2 μg plasmid DNA in opti-MEM (31985-070, Invitrogen) containing 2 μL Neofect™ DNA transfection reagent (TF20121201) following the protocol for 24–48 h.

### Fibronectin coating of coverslips

The 1 mg/mL fibronectin stock is diluted to 10 μg/mL with PBS. 50 μL diluted fibronectin solution is added to the φ 12 mm coverslips in 12-well plate, followed by incubation at 37 °C for 30 min. After incubation, the excess fibronectin is discarded and the coverslips are washed with PBS.

### Antibodies and reagents

The following antibodies were used in this study: rabbit anti-Myo19 (HPA059715, 1:1000 for western blotting) from Sigma-Aldrich; rabbit anti-Mic60 (A2751, 1:3000 for western blotting), anti-Metaxin2 (A7958, 1:1000 for western blotting) and rabbit anti-Sam50 (A3401, 1:3000 for western blotting, 1:400 for PLA assay) from ABclonal; mouse anti-Mic60 (sc-390707, 1:200 for immunofluorescence staining) from Santa Cruz Biotechnology; rabbit anti-GFP (598, 1:1000 for western blotting, 1:200 for immunofluorescence staining) and mouse anti-GFP (MO48-3, 1:200 for immunofluorescence staining) from MBL; anti-mouse (sc-516102, 1:4000) and anti-rabbit (sc-2004, 1:4000) horseradish peroxidase (HRP)-conjugated secondary antibodies from Santa Cruz Biotechnology; Alexa Fluor488- or Alexa Fluor 555-conjugated secondary antibody from ThermoFisher Scientific. For reagents, MitoTracker^TM^ Red CMXRos (M7512), MitoTracker^TM^ Green FM (M7514), TMRE (T669), TMRM (T668) and Prolong Diamond Antifade with DAPI (P36962) were purchased from Thermo Fisher Scientific. Rotenone (HY-B1756), FCCP (HY-100410) and 2-Deoxy-D-glucose (HY-13966) were purchased from MedChemExpress.

### Plasmid constructions

The full length human Myo19, Sam50, MTX2, MTX3 were cloned from the MDA-MB-231 cell-extracted cDNA library and was subcloned into lentiviral vector (Plvx-ac-GFP-N1) with C-terminal tagged EGFP using an ABclonal MultiF Seamless Assembly kit (RK21020, ABclonal). The Myo19 truncations were amplified and cloned into the same vectors. The FL-Myo19 (1-970 aa), ΔTail (1-823 aa) and ΔMotor-3IQ (824-970 aa) were constructed.

### Generation of knockout and knockdown cells

For gene knockout, the following sgRNAs in LentiCRISPR-V2 (#52961, Addgene) were used in MDA-MB-231 cells. After puromycin selection, the single cell clones were cultured and verified by sequencing and western blotting.

For Myo19 knockout:

sgRNA-1: 5'-ATGGTACTCTCTCATTAGCT-3';

sgRNA-2: 5'-GACACATTCTACACCAATGC-3'.

Sequencing primer:

Myo19-seq-F: 5'- TATGGGCCTCCCATTCCTCA-3';

Myo19-seq-R: 5'- TCCCCACTTTGCACCTTCTG-3';

For Miro1 knockout:

sgRNA-Miro1-1: 5'-GGCTGCCTTTAATATTGGTT-3';

sgRNA-Miro1-2: 5'-TCTGGTGGAATATAGTAGTA-3';

Sequencing primer:

Miro1-seq-F: 5'-GGGGCTCAACCTGACTGTAG-3';

Miro1-seq-R: 5'- TACCTCCTTCTCCTCTGGGC-3';

For Miro2 knockout:

sgRNA-Miro2-1: 5'- TTCTCCGGGGTGACGTCCGC-3';

sgRNA-Miro2-2: 5'- ATGGTGATCTCCTCCGCGCG-3';

Sequencing primer:

Miro2-seq-F: 5'- CCCGTCCCTTAATCGGCTG-3';

Miro2-seq-R: 5'- CCACTGGACTGAGCGGTG-3';

For Myo19 and Sam50 knockdown in MDA-MB-231 cells, the following shRNAs were cloned into pLKO.1 vector. After lentivirus infection and puromycin selection, the pooled cells were verified by the western blotting.

scramble: 5'-AACGCTGCTTCTTCTTATTTA-3';

shMyo19: 5'-TACAGCATTACAGGCTTTAAT-3'; 5'-GTGTATGGATTTGAATCATTT-3';

shSam50: 5'-GGTCATCGATTCTCGGAAT-3'.

For Mic60 knockdown, the siRNA targeting to Mic60 was purchased from Life Technologies (MSS293683).

### Generation of Myo19-EGFP knock-in cells

The following sgRNAs were cloned into lentiCRISPR-V2 vector. The donor templates contained left homologous arm (800 bp before the stop code), EGFP/mcherry sequence and right homologous arm (800 bp after the stop code). The homologous arms were amplified from the MDA-MB-231 cell-extracted genome DNA. To generate the knock-in cells, sgRNA and relative donor template plasmid were co-transfected to the cells. After 24 h, puromycin was used to eliminate the puromycin-sensitive cells. The fluorescence positive cells were acquired by Fluorescence Activated Cell Sorting (FACS) and the single cell clones were cultured and verified by PCR and western blotting.

sgRNA for Myo19 C-terminal knock-in in MDA-MB-231 cells:

sgRNA: 5'-TTGTGGAAACAAAGGCACCA-3'.

Primers for screening Myo19-EGFP knock-in cells:

Myo19-knockin-F: 5'-AAGTCTCCACTGCGGTATGC-3';

Myo19-knockin-R: 5'-GGGATCCCCAGTGTAGTGAC-3'

### Western blotting

For western blotting, cells were washed with DPBS (Dulbecco’s phosphate-buffered saline, containing no Ca^2+^ and Mg^2+^) once and lysed with appropriate volumes of RIPA buffer (50 mM Tris-HCl, pH 8.0, 150 mM NaCl, 1% Triton X-100, 0.5% Na-deoxycholate, 0.1% SDS, 1 mM EDTA and protease inhibitor cocktail) for 10 min on ice. The cell lysis was centrifuged at 13,572 *g* for 10 min at 4 °C, and the supernatants were collected. Then, 5× SDS loading buffer was added to the supernatants and the mixtures were boiled for 10 min at 95 °C. Protein samples were separated on 10% SDS-PAGE gels and transferred onto NC membranes by wet electrophoretic transfer, followed by first antibodies incubation at 4 °C overnight or room temperature for 2 h, and second antibodies incubation at room temperature for 1 h. The X-ray films were used to detect and record the band intensities. The fixed X-ray films were scanned to obtain digital images. The images were processed by ImageJ software (https://imagej.nih.gov/ij/).

### Co-immunoprecipitation (Co-IP)

Cells were lysed with IP lysis buffer (150 mM NaCl, 25 mM Tris-HCl, 0.5% NP-40, pH 7.4 and protease inhibitor cocktail) for 30 min with gentle rotating. The cell lysis was centrifuged at 13,572 *g* for 10 min at 4 °C to remove the insoluble components. The supernatants were added with antibodies (1 μg antibody per 500 μg cell lysis) and rotated at 4 °C overnight. Then the protein A/G beads (P2012; Beyotime) were washed with IP lysis buffer and added to the antibodies-supernatants mixtures followed by 3 h rotating at 4 °C. Finally, the beads were washed three times with lysis buffer and boiled in 1x SDS sample buffer. To detect the interaction proteins, the samples were separated by 10% SDS-PAGE gels and analysed by western blotting. To analyse protein interactome, the samples were separated by 10% SDS-PAGE gels and the target gels were collected for mass spectrum.

### Mass spectrum (MS)

For protein identification, the Coomassie-stained total aggregated proteins of each sample were cut out of the gel and destained with a solution of 100 mM ammonium bicarbonate in 50% acetonitrile. After dithiothreitol reduction and iodoacetamide alkylation, the proteins were digested with porcine trypsin (Sequencing grade modified; Promega, Madison, WI) overnight at 37 °C. The resulting tryptic peptides were extracted from the gel pieces with 80% acetonitrile, 0.1% formic acid (FA). The samples were dried in a vacuum centrifuge concentrator at 30 °C and resuspended in 10 μl 0.1%FA.

Using an Easy-nLC 1200 system, 5 μl of sample were loaded at a speed of 0.3 μl/min in 0.1% FA onto a trap column (C18, Acclaim PepMap TM 100 75 μm × 2 cm nanoViper Thermo) and eluted across a fritless analytical resolving column (C18, Acclaim PepMap TM 75 μm × 25 cm nanoViper RSLC Thermo) with a 75-min gradient of 4 to 90% LC-MS buffer B (LC-MS buffer A includes 0.1% formic acid; LC-MS buffer B includes 0.1% formic acid and 80% ACN) at 300 nl/min.

Peptides were directly injected into a Thermo Orbitrap Exploris 480 with FAMIS pro using a nano-electrospray ion source with electrospray voltages of 2.2 kV. Full scan MS spectra were acquired in the Orbitrap mass analyzer (m/z range: 350–1500 Da) with the resolution set to 60,000 (FWHM) at m/z 200 Da. Full scan target was 300% with a maximum IT of 30 ms. All data were acquired in profile mode using positive polarity. MS/MS spectra data were acquired in the Orbitrap as well with a resolution of 15,000 (FWHM) at m/z 200 Da and AGC target value for fragment spectra was set at 100% with a maximum IT of 22 ms. Isolation width was 1.6 m/z. Normalized collision energy was set at 30%. FAIMS CV were set as −45 V and −65 V. The MS data were aligned with UniProtKB/Swiss-Prot reviewed human proteome database.

### Proximity ligation assay (PLA)

PLA was performed with Duolink kits from Sigma-Aldrich. Myo19-EGFP knock-in cells were fixed with −20 °C pre-chilled 100% methanol at −20 °C for 15 min. After 3 times PBS washing, the cells were permeabilized with 0.3% Triton X-100 in PBS for 5 min. Commercial blocking solution was added to the samples and incubated for 1 h at room temperature. After blocking, the cells were incubated with the diluted antibodies for 1 h at room temperature followed by 3 times PBS washing. The PLUS and MINUS PLA probes were mixed and diluted (1:5) in antibody diluent and incubated with samples for 30 min at 37 °C. Then, the samples were washed in 1× Wash Buffer A for 5 min twice. The ligase was diluted (1:40) in ligation buffer (1:5 diluted in H_2_O) and incubated with samples for 30 min at 37 °C, followed by washing with 1× Wash Buffer A for 2 min twice. The polymerase was diluted (1:80) in amplification stock (1:5 diluted in H_2_O) and incubated with samples for 100 min at 37 °C. The samples were then washed in 1× Wash Buffer B for 10 min twice, followed by another washing in 0.01× Wash Buffer B for 1 min. Finally, the samples were mounted with Prolong Diamond Antifade with DAPI for 30 min at room temperature. For positive control of PLA experiments, anti-tyrosinated α-tubulin and anti-α-tubulin antibodies were used to identify the positive signals. For negative control of PLA experiments, anti-Sam50 or anti-GFP antibodies were used alone.

### Immunofluorescence staining and imaging analysis

Myo19-EGFP knock-in cells were plated on acid-washed coverslips coated with 10 μg/mL fibronectin for 1 h. Cells were stained with 1 μM Mitotracker^TM^ Red CMXRos at 37 °C for 20 min, followed by fixation with −20 °C pre-chilled 100% methanol at −20 °C for 15 min. Then the cells were permeabilized with 0.3% Triton X-100 in PBS for 5 min, washed with PBS once for 5 min and blocked with 10% bovine serum albumin (BSA) for 1 h at room temperature. Antibodies were diluted in PBS and incubated for 1 h at room temperature. After 3 times washing with PBS, the coverslips were incubated with secondary antibodies for 45 min at room temperature. After another 3 times washing in PBS, the coverslips were mounted with Prolong Diamond Antifade with DAPI. Images were captured using a SP8 LIGHTNING confocal microscope. Image J was used to process all images.

### EM/FIB-SEM

Cells were seeded on the ACLAR® Films (50425) in 12-well plates and cultured for 12 h before fixation. Then cells were washed with 37 °C PB buffer (0.2 mol/L NaH_2_PO_4_ and Na_2_HPO_4_, pH = 7.2–7.4), and immediately fixed with PB buffer containing 2% PFA and 2.5% glutaraldehyde at room temperature for 1 h and 4 °C overnight. After post-fixation in 1% osmium tetroxide and pre-embedding staining with 1% uranyl acetate, samples were dehydrated and embedded in SPI-Pon 812 resin. EM images were acquired using Jeol JEM-1400 electron microscope operated at 80 kV.

For FIB-SEM, images were acquired at 2 kV with the ICD detector (pixel size x/y 3 nm) in a continuous mill and acquire mode. A 790 pA current was applied to remove 5 nm between every image. Data processing was performed on Amira software.

### Hessian-SIM

The Myo19-EGFP knock-in MDA-MB-231 cells were plated on the glass bottom dish and cultured for 12 h before imaging. To label mitochondria, cells were incubated with 200 nM MitoTracker™ Red CMXRos in high-glucose DMEM at 37 °C for 15 min followed by 3 times PBS washing. Images were captured and processed on a previously reported Hessian-SIM^45^.

To estimate the theoretical distribution rate of randomly distributed dots on cristae (expected value), we used ImageJ and set threshold to determine boundaries of MitoTracker^TM^ Red CMXRos stained-cristae (ImageJ->Image->Adjust->Threshold), and used line scan to quantify the ratios of pixels with positive fluorescence intensity to overall pixels.

### Ultrastructure expansion microscopy(U-ExM)

U-ExM procedure was carried out as described by Gambarotto et al., 2021. Briefly, WT and Myo19-EGFP knock-in MB-MDA-231 cells were seeded on fibronectin-coated 12 mm coverslips for 12 h before fixation using 4% PFA for 10 min at room temperature. After fixation, the coverslips were immersed in a anchoring solution containing 0.7% formaldehyde (Sigma, F8775) and 1% acrylamide for 5 h at 37 °C. Next, each coverslip was placed on top of one drop of gelling solution (23% (w/v) sodium acrylate (Sigma, 408220), 10% acrylamide, 0.1% N,N'-methylenebisacrylamide, 0.5% ammonium persulfate and 0.5% N,N,N',N'-TEMED) with cells facing down. After incubated on ice for 5 min, the gelation process was fully initiated and completed at 37 °C for 1 h. Gels were then detached from the coverslips and denatured in denaturation buffer (200 mM SDS, 200 mM NaCl and 50 mM Tris-BASE, pH = 9.0) for 20 min at 90 °C. First round of expansion was performed in ddH2O for the whole night and followed by primary and secondary antibody labelling. Prior to confocal imaging, gels were completely expanded through one more round of expansion and mounted on poly-L-lysine-coated confocal dishes.

### Protein expression and purification

For recombinant protein expression in E. coli BL21 (DE3) cells, DNA fragments were cloned into the pET28a (+) vector according to the previously study^68^. Full length Myo19 and Myo19^824-970^ were cloned into pET28a (+) vector with N-terminal MBP tag and C-terminal Flag tag. Metaxin2, Metaxin3, Sam50 and its truncations were cloned into pET28a (+) with N-terminal 6xHis-MBP tag. The related plasmids were transformed into E. coli BL21 (DE3) cells and protein expression was induced with 100 μM IPTG at 16 °C. To determine whether the protein was expressed in the inclusion body, the cells expressed proteins were lysed in PBS supplied with 1% Triton-X100 and 1 mM phenylmethanesulfonylfluoride (PMSF) using ultrasonic cell crusher and centrifuged at 13,572 *g* for 10 min. The supernatant and pellet were collected for western blotting. For, Myo19^824-940^, Metaxin2 and Metaxin3 purification, the cells expressed proteins were lysed in 20 mM Tris, pH 7.4, 140 mM NaCl, 5 mM KCl, 10% glycerol, 1 mM Tris (2-carboxyethyl) phosphine (TCEP) and 1 mM phenylmethanesulfonylfluoride (PMSF) using ultrasonic cell crusher and centrifugation at 48,380 *g* for 30 min. The supernatant was applied to a Ni-IDA beads (Smart-Lifesciences) and washed with buffer containing 20 mM Tris, pH 8.0, 500 mM NaCl, 1% glycerol and 0.5 mM TCEP with appropriate concentrations of imidazole. After that, proteins were eluted with elution buffer containing 20 mM Tris, pH 8.0, 500 mM NaCl, 1% glycerol, 0.5 mM TCEP and 300 mM imidazole. For Sam50 and its truncations purification, 1% Triton-X100 was added and TCEP was removed from the purification procedures. Proteins were concentrated by centrifugation at 3000 *g* at 4 °C until reaching the volume of 500 μL using 10 kDa concentrator (UFC9010, Sigma-Aldrich), and then loaded onto a Superdex200 Increase 10/300 (GE Healthcare) equilibrated with gel filtration buffer containing 20 mM Tris, pH 8.0, 150 mM NaCl, 1% glycerol and 1 mM TCEP. Peaks containing proteins were collected and evaluated with Coomassie blue staining of SDS–PAGE gels.

### Pull-down assay

Purified Metaxin2, Metaxin3, Sam50 and its truncations were incubated with Myo19^824-970^ and Flag antibody in IP lysis buffer (150 mM NaCl, 25 mM Tris-HCl, 0.5% NP-40, pH 7.4 and protease inhibitor cocktail) at 4 °C overnight. Then the protein A/G beads (P2012; Beyotime) were washed with IP lysis buffer and added to the antibodies-supernatants mixtures followed by 3 h rotating at 4 °C. Finally, the beads were washed three times with lysis buffer and boiled in 1x SDS sample buffer. To analyse protein interaction, the samples were separated by 10% SDS-PAGE gels and the proteins were detected using western blotting.

### ATP concentration assay

Cellular ATP was measured using ATP Assay Kit (S0026, Byotime) by a 96-well plate reader. BCA protein assay kit (P0012, Beytime) was used to adjust protein abundance

### Oxygen consumption and glycolytic analysis

The oxygen consumption rate (OCR) and the extracellular acidification rate (ECAR) were evaluated using a Seahorse XF96 Analyzer (Seahorse Bioscience) as described previously^69^.

Briefly, 2 × 10^4^ MDA-MB-231 WT and mutant cells were seeded on the XF96 cell plate and cultured for 12 h before the assay. OCR were measured in XF assay medium containing 4.5 g/L glucose, 2 mM glutamine and 1 mM sodium pyruvate, followed by the sequential addition of 1 μM oligomycin, 1 μM FCCP, and 1 μM rotenone. ECAR was measured in XF assay medium containing 2 mM glutamine, followed by the sequential addition of 4.5 g/L glucose, 1 μM oligomycin and 50 mM 2-DG. The basal OCR and ECAR values corresponded to the difference before and after oligomycin or glucose addition. The maximal OCR was calculated by OCR after FCCP addition minus OCR after oligomycin addition. The maximal ECAR was calculated by ECAR after oligomycin minus ECAR before glucose addition.

### Theoretical model

We introduced thermal strain to simulate the synthesis or growth of the mitochondrial membrane. Here we considered a two-dimensional system. The relationship between the total geometrical strain $\left\{\varepsilon \right\}$ and the displacement reads

1 $$\left\{\varepsilon \right\}=\left\{\begin{array}{c}{\varepsilon }_{x}\\ {\varepsilon }_{y}\\ {\gamma }_{{xy}}\end{array}\right\}=\left[\begin{array}{cc}\frac{\partial }{\partial x} & 0\\ 0 & \frac{\partial }{\partial y}\\ \frac{\partial }{\partial x} & \frac{\partial }{\partial y}\end{array}\right]\left\{\begin{array}{c}u\\ v\end{array}\right\}.$$

where ${\varepsilon }_{x}$, ${\varepsilon }_{y}$, and ${\gamma }_{{xy}}$ are the components of $\{\varepsilon \}$; $u$ and $v$ denote the displacement in the x and y direction. The total geometrical strain $\{\varepsilon \}$ can be decomposed as

2 $$\{\varepsilon \}=\{\varepsilon \}_{E}+\{\varepsilon \}_{G}$$

where $\{\varepsilon \}_{E}$ is the elastic strain and $\{\varepsilon \}_{G}$ is the growth strain induced by synthesis of mitochondrial membrane. Only the elastic strain contributes to the stress. Therefore, the stress-strain relationship of in the system can be expressed as:

3 $$\{\sigma \}=[D]\left(\{\varepsilon \}-\{\varepsilon \}_{G}\right)$$

where $\{\sigma \}$ is the stress and $[D]$ is the matrix of stiffness. Following thermo-elasticity^70^, we use the thermal expansion to simulate the in-plane growth of mitochondrial membrane, that is, $$\{\varepsilon \}_{G}=\left[\begin{array}{ccc}\alpha \varDelta T & 0 & 0\end{array}\right]$$, where $\alpha$ denotes the thermal expansion coefficient and $\varDelta T$ is the change of temperature. In a finite element formula, $\varDelta T=[N]_{G}\{\varDelta T\}^{e}$,$$\{\begin{array}{c}u\\ v\end{array}\}=[N]\{\delta \}$$ where $[N]$ is the shape function and $\{\delta \}$ is the nodal displacement. The elastic strain energy ${U}^{e}$ of an element can be described as

4 $${U}^{e}= \frac{1}{2}{\int }_{{V}^{e}}{([B]\{\delta \}^{e})}^{T}[D][B]\{\delta \}^{e}{dV}+\frac{1}{2}{\int }_{{V}^{e}}\{\varepsilon \}_{G}^{T}[D]\{\varepsilon \}_{G}{dV}\\ -{\int }_{{V}^{e}}{([B]\{\delta \}^{e})}^{T}[D]\{\varepsilon \}_{G}{dV}=\frac{1}{2}{\{{\delta }^{e}\}}^{T}{[k]}^{e}\{{\delta }^{e}\}-{\left\{{\delta }^{e}\right\}}^{T}\{Q\}_{G}^{e}+C$$

where

5 $${[k]}^{e}={\int }_{{V}^{e}}{[B]}^{T}[D][B]{dV}$$

6 $${\left\{Q\right\}}_{G}^{e}={\int }_{{V}^{e}}{[B]}^{T}[D]\{\varepsilon \}_{G}{dV}$$

7 $$C=\frac{1}{2}{\int }_{{v}^{e}}{\{\varepsilon \}}_{G}^{T}[D]{\{\varepsilon \}}_{G}dV$$

Here $\{B\}$ represents the matrix of strain. Without external loading, the principle of potential energy minimization yields

8 $$\delta U=\delta (\sum {U}^{e})=0$$

9 $${U}^{e}=\frac{1}{2}\{{\delta }^{e}\}^{T}{[k]}^{e}\{{\delta }^{e}\}-\{{\delta }^{e}\}^{T}\{Q\}_{G}^{e}+C$$

10 $$[K]\{\delta \}=\{Q\}_{G}$$

where

11 $$[K]=\sum {[k]}^{e}$$

12 $$\{Q\}_{G}=\sum {\{Q\}}_{G}^{e}$$

Here [*K*] is the matrix of total structural stiffness and {*Q*}_*G*_ is the vector of total growth load. When there are other external loads on the structure, the external loads can be superimposed with the growth loads.

Finite element simulations: we implement our finite element method in Abaqus (6.14). The mitochondrial membrane was discretized to two-dimensional deformable shell elements in a Cartesian rectangular coordinate system (o-xy) (Supplementary Fig. 1B). The material properties were linearly elastic. The inner membrane was allowed to grow only in the horizontal axis direction in the local coordinate system. The geometrical parameters of the model were determined according to the experimental measurement measurements. The system was meshed by 14580 CPS8R elements and a mesh sensitivity analysis was made to ensure that the element sizes did not interfere with the numerical results. To better capture the deformation and instability, the fine meshes were used in the region of membrane growth. Small geometrical imperfections were introduced to trigger elastic instability. We ignored the relative movement of mitochondria and cell sap. The outer mitochondrial membrane was imposed as fixed displacement constraints. For the inner membrane of mitochondria, we imposed corresponding fixed constraints within the scope of Myo19 at Cristea Junctions (CJs).

### Statistics and reproducibility

Experiments were performed in duplicate and repeated at least two times except for main Figs. 2i, 5a, Supplementary Figs. 2i, 3d, 3e, 6. Data are mean ± SD as indicated in the figure legends and supplementary figure legends. One-way ANOVA analysis with multiple comparisons test, unpaired two-sided *t*-test and Chi-square test were performed with GraphPad Prism 7.0.

### Reporting summary

Further information on research design is available in the Nature Research Reporting Summary linked to this article.

## Supplementary information

Supplementary Information

Description of Additional Supplementary Files

Supplementary Movie 1

Reporting Summary

## Data Availability

Data supporting the findings of this work are available within the paper and its Supplementary Information files. The mass spectrometry proteomics data have been deposited to the ProteomeXchange Consortium via the PRIDE^71^ partner repository with the dataset identifier PXD033198. Source data are provided with this paper.

## Source data

Source Data
