# Supplementary material for: Mechanical instability generated by Myosin 19 contributes to mitochondria cristae architecture and OXPHOS
Source: Nat Commun. 2022 May 13;13:2673. doi: 10.1038/s41467-022-30431-3 (PMC9106661; doi:10.1038/s41467-022-30431-3)
Supplement: Supplementary file 1 — Supplementary Information [file 41467_2022_30431_MOESM1_ESM.pdf]

Supplementary information

Title: Mechanical instability generated by Myosin 19 contributes to mitochondria cristae architecture and OXPHOS

Shi et al., Nature Communications, 2022

This file contains:

Supplementary Figures (1 to 7)

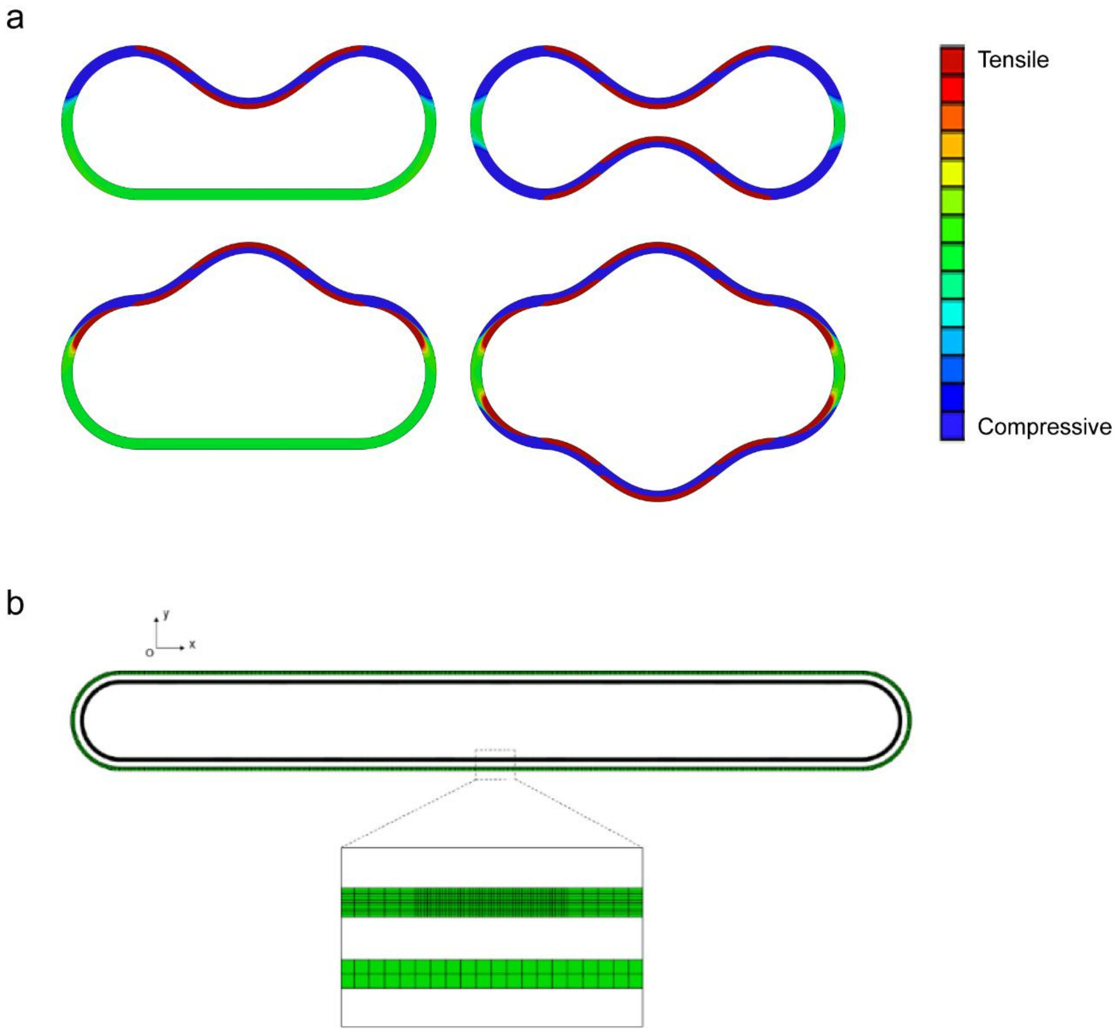

Supplementary Figure 1: Mechanical modelling of abnormal mitochondria morphology.

a. Mechanical modelling of abnormal mitochondria morphology. Mitochondria with low density of CJs underwent low tethering force and exhibited abnormal mitochondria morphology. Color indicates membrane stress. **b.** The finite element model of the mitochondrial membranes and meshes of the membranes.

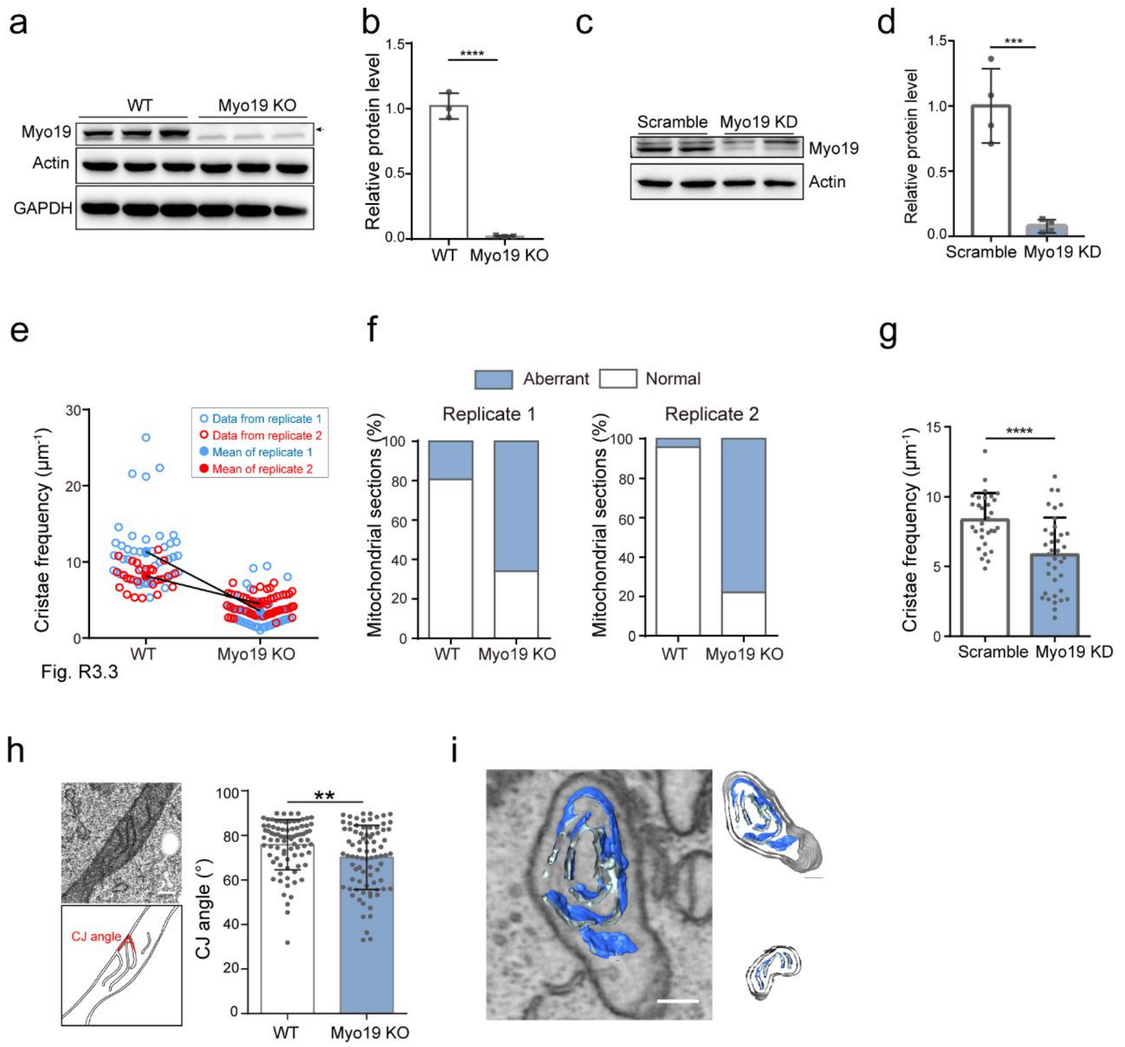

Supplementary Figure 2: Loss of Myo19 impaired mitochondrial cristae structure.

a. Immunoblotting of WT and Myo19 KO cells. β-actin and GAPDH were used as loading controls. **b.** Quantification of relative protein level in Supplementary Fig. 2a. Data are shown as mean ± SEM. N_{WT}=3, $N_{Myo19\ KO}$=3 independent samples. ****, *P*<0.0001. Significance (*P*-value) was evaluated by two-sided *t*-test. **c.** Immunoblotting of WT and Myo19 KD cells. β-actin and GAPDH were used as loading controls. **d.** Quantification of relative protein level in Supplementary Fig. 2c. Data are shown as mean ± SEM. $N_{scramble}$=4, $N_{Myo19\ KD}$=4 independent samples. ***, P=0.0007. Significance (*P*-value) was evaluated by two-sided *t*-test. **e.** Super-plot of the cristae frequency of WT and Myo19 KO cells on two replicates. ****, P<0.0001. Data are shown as mean ± SD. Significance (*P*-value) was evaluated by two-sided *t*-test. **f.** Super-plot of the percentage of cristae morphology on two replicates. **g.** Quantification of the cristae frequency of scramble and

Myo19 knockdown (Myo19 KD) cells. ****, P<0.0001. Data are shown as mean±SD. $N_{scramble}$=31, $N_{Myo19\ KD}$=37 mitochondria. Significance (*P*-value) was evaluated by two-sided *t*-test. **h.** Left: representative EM image of mitochondria cristae with aberrant geometric angles. The outlines of cristae are delineated with black lines and the CJ angle is marked in red. Scale bar: 200 nm. Right: quantification of CJ angle on EM images. Data are shown as mean ± SD. N_{WT}=83, $N_{Myo19\ KO}$=82 CJs. **, *P* =0.0047. Significance (*P*-value) was evaluated by two-sided *t*-test. **i.** Representative reconstructed FIB-SEM images of onion-shaped Myo19 KO mitochondria. Scale bar: 200 nm. Source data are provided as a Source Data file.

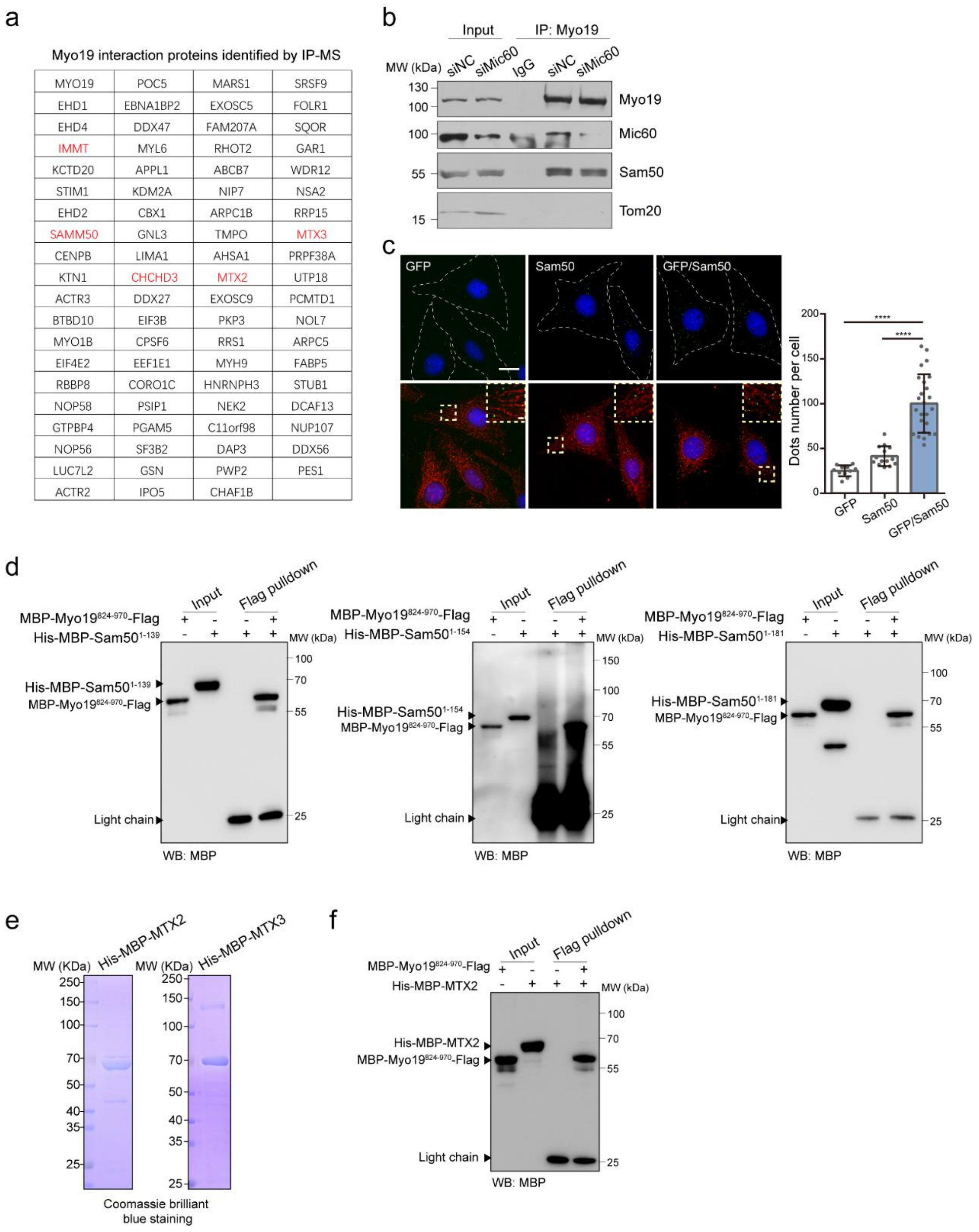

Supplementary Figure 3: Myo19 interacting proteins on mitochondria.

a. The list of Myo19 interacting proteins from IP-MS data. Proteins in SAM-MICOS complex were highlighted with red font. **b.** Co-immunoprecipitation of Myo19 with endogenous Sam50 and Mic60 in MDA-MB-231 cells transfected with small interfering RNA of Mic60 (siMic60) and scrambled negative control siRNA (siNC). Tom20 was used as loading control. The immunoprecipitates are blotted as indicated. **c.** Left: representative images of *in situ* proximity ligation assay (PLA) between Myo19 and

Sam50 in MDA-MB-231 Myo19-EGFP knock-in cells. The cell outlines are delineated with yellow dash lines. PLA positive dots are shown in green. Tom20 (Red) was used to label mitochondria and DAPI (blue) to nuclei. The white dashed line indicates cell outline and yellow dashed boxes are zoomed in at the upper right. Scale bar: 20 μm (2 μm in magnification). Right: quantification of PLA positive dots per cell. Data are shown as mean ± SD. N_{GFP}=11, N_{Sam50}=14, $N_{GFP/Sam50}$=25 dots. ****, *P* <0.0001. Significance (*P*-value) was evaluated by two-sided *t*-test. **d.** In vitro pull-down assay of the purified MBP-Myo19$^{824-970}$-Flag and His-MBP-Sam50$^{1-139}$ / Sam50$^{1-154}$ / Sam50$^{1-181}$. **e.** Coomassie blue staining of purified protein as indicated. **f.** In vitro pull-down assay of the purified MBP-Myo19$^{824-970}$-Flag and His-MBP-MTX2. Source data are provided as a Source Data file.

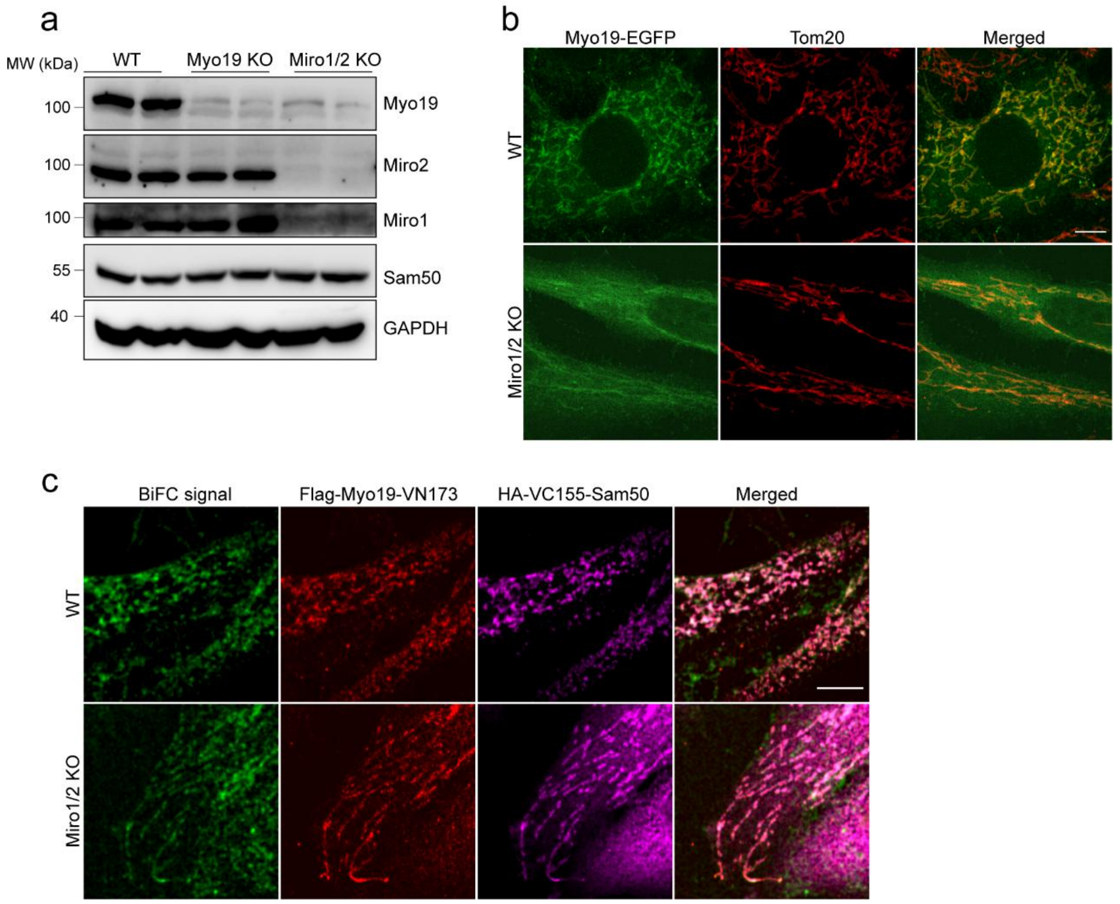

Supplementary Figure 4: The role of Miro1/2 in the interaction of Myo19 and SAM/MICOS complex.

a. Immunoblotting of indicated proteins in WT, Myo19 KO and Miro1/2 DKO MDA-MB-231cells. **b.** Representative images of WT and Miro1/2 DKO cells expressing full length Myo19-EGFP. Scale bar: 10 μm. **c.** Bimolecular fluorescence complementation (BiFC) analysis of Myo19 and Sam50 in WT and Miro1/2 DKO cells. Cells were co-transfected with Flag-Myo19-VN173 and HA-VC155-Sam50, followed by immunofluorescence staining of Flag and HA antibodies to visualize Myo19 (red) and Sam50 (magenta), respectively. BiFC fluorescence was pseudo-colored green. Scale bar: 10 μm. Source data are provided as a Source Data file.

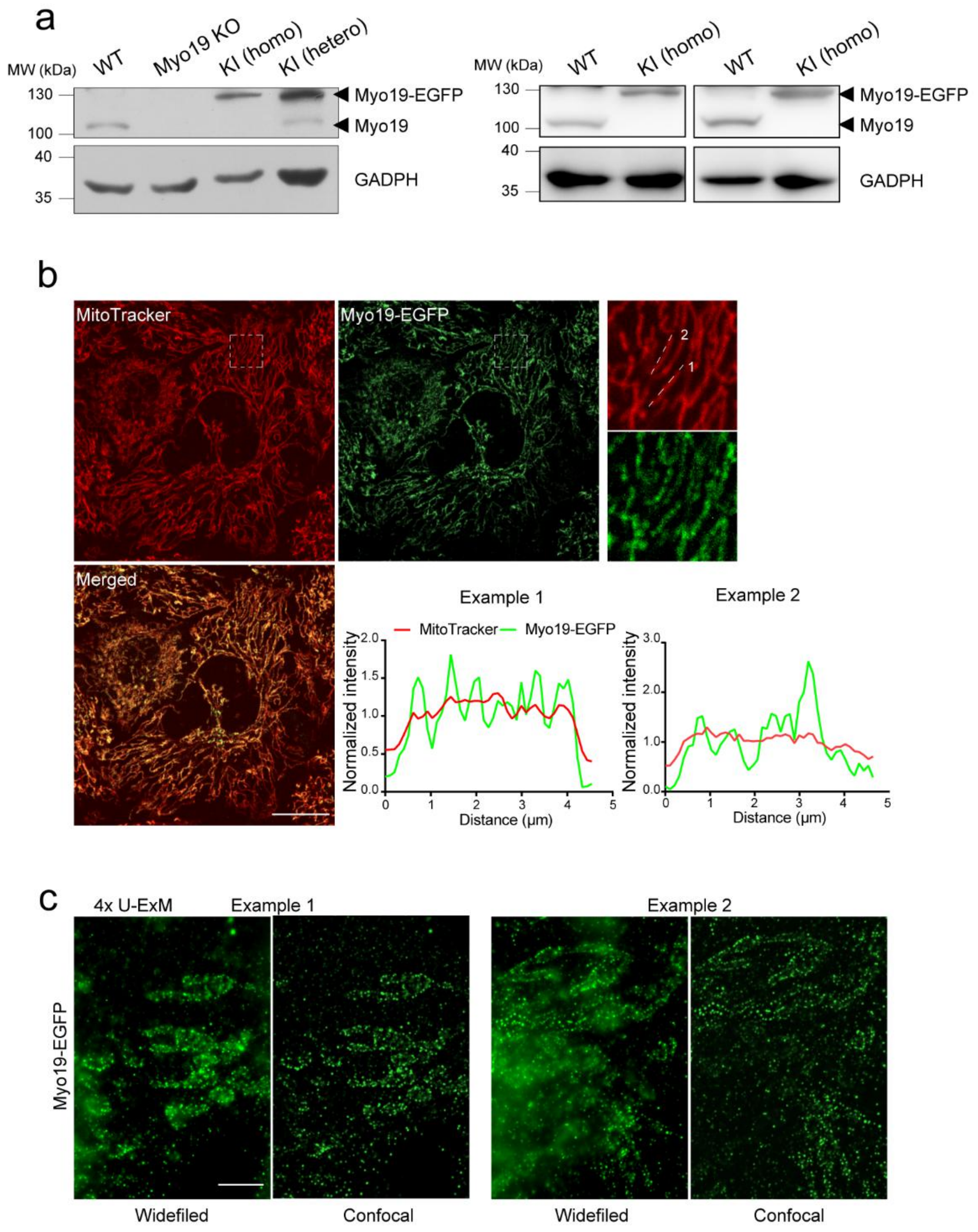

Supplementary Figure 5: Myo19-EGFP knock-in cells.

a. Immunoblotting of homozygous and heterozygous MDA-MB-231 Myo19-EGFP knock-in cells. GAPDH was used as loading control. KI (homo) indicates homozygous Myo19-EGFP knock-in cells. KI (hetero) indicates heterozygous Myo19-EGFP knock-in cells. **b.** Left: representative images of MDA-MB-231 Myo19-EGFP knock-in cells stained with 200 nM MitoTracker^{TM} Red CMXRos (Mito) for 15 min. Scale bar: 20 μm. Right: quantification of normalized fluorescent intensity by line scanning analysis. **c.** Representative images of MDA-MB-231 Myo19-EGFP knock-in cells stained with GFP

antibody and the sample was prepared by U-ExM method. The expansion factor was about 4.5. Scale bar: 10 μm. Source data are provided as a Source Data file.

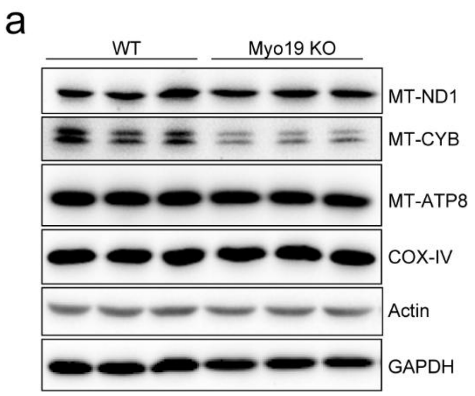

Supplementary Figure 6: OXPHOS-related proteins upon Myo19 depletion.

a. Immunoblotting of mitochondria proteins (MT-ND1, MT-CYB, MT-ATP8, COX-IV) in WT and Myo19 KO cells. β-actin and GAPDH were used as loading controls. Source data are provided as a Source Data file.

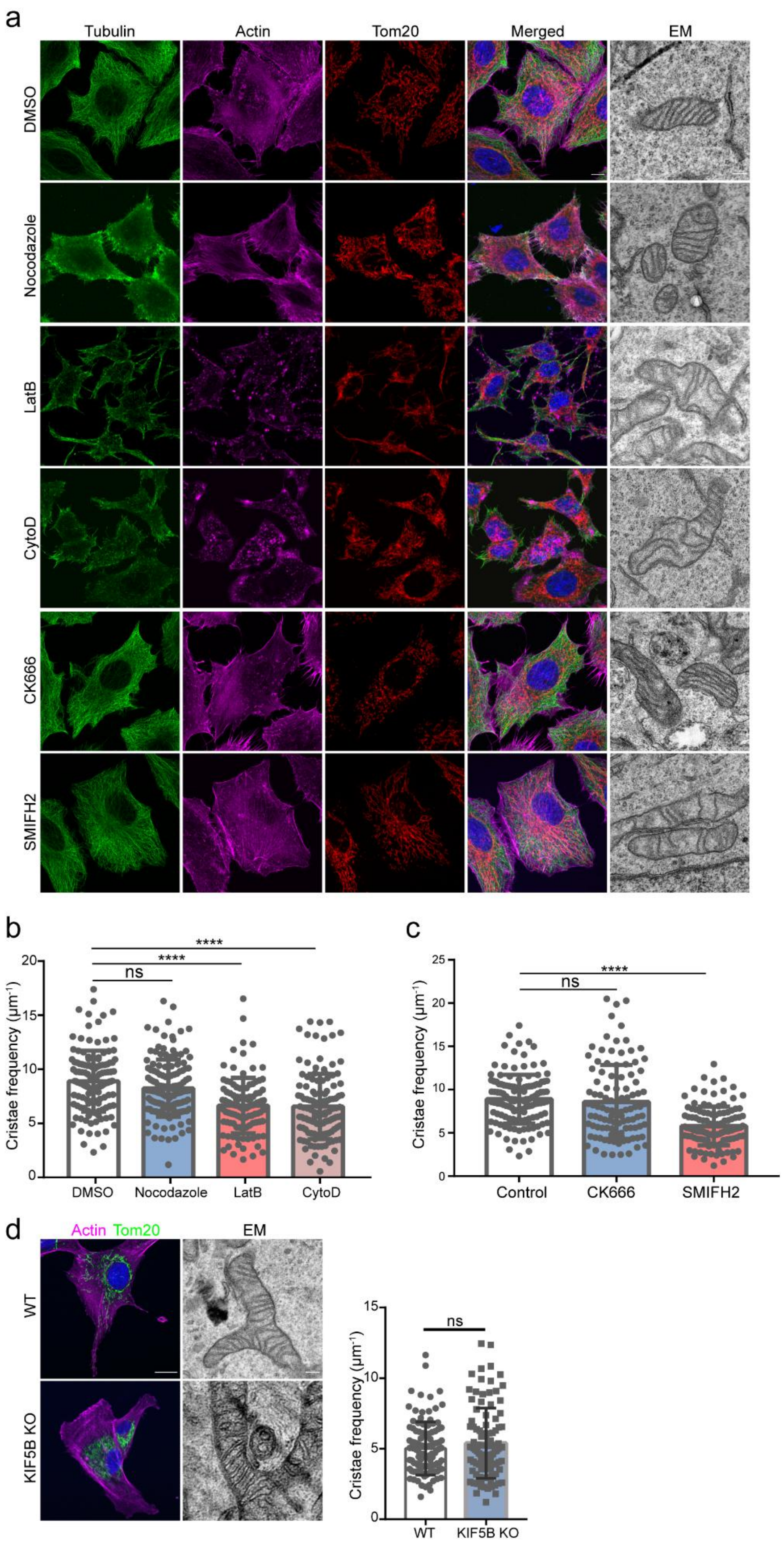

Supplementary Figure 7: The effect of cytoskeleton on cristae structure regulation

a. Representative IF and EM images of cells treated with nocodazole (8 μM, 0.5 h),

latrunculin B (LatB, 0.5 μM, 1 h), cytochalasin D (CytoD, 1 μM, 1 h), CK666 (100 μM, 6 h), or SMIFH2 (15 μM, 6 h). Scale bar: IF: 20 μm, EM: 200 nm. **b.** Quantification of the overall cristae frequency on EM images in Fig. A. Data are shown as mean ± SD. N_{DMSO}=130, $N_{Nocodazole}$=138, N_{LatB}=108, N_{CytoD}=123 mitochondria. ****, P <0.0001. Significance (*P*-value) was evaluated by two-sided *t*-test. **c.** Quantification of the overall cristae frequency on EM images in Fig. A. Data are shown as mean ± SD. N_{DMSO}=130 N_{CK666}=119, N_{SMIFH2}=119 mitochondria. ****, P <0.0001. "ns" indicates no significance. Significance (*P*-value) was evaluated by two-sided *t*-test. **d.** Left: representative IF and EM images of WT and KIF5B knockout cells. Scale bar: IF: 20 μm, EM: 200 nm. Right: quantification of the overall cristae frequency on EM images. Data are shown as mean ± SD. N_{WT}=107 $N_{KIF5B\ KO}$=101 mitochondria. "ns" indicates no significance. Significance (*P*-value) was evaluated by two-sided *t*-test. Source data are provided as a Source Data file.
