# Supplementary material for: Mechanical instability generated by Myosin 19 contributes to mitochondria cristae architecture and OXPHOS
Source: Nat Commun. 2022 May 13;13:2673. doi: 10.1038/s41467-022-30431-3 (PMC9106661; doi:10.1038/s41467-022-30431-3)
Supplement: Supplementary file 2 — Description of Additional Supplementary Files [file 41467_2022_30431_MOESM2_ESM.pdf]

File name: Supplementary Movie 1

Description: **The simulations of the ridge formation.** The simulation of the ridge formation based on thermal expansion using Abaqus 6.14.
