# Supplementary material for: Mechanical instability generated by Myosin 19 contributes to mitochondria cristae architecture and OXPHOS
Source: Nat Commun. 2022 May 13;13:2673. doi: 10.1038/s41467-022-30431-3 (PMC9106661; doi:10.1038/s41467-022-30431-3)
Supplement: Supplementary file 4 — Reporting Summary [file 41467_2022_30431_MOESM4_ESM.pdf]

nature research

Corresponding author(s): DBPR, NCOMMS-21-06531B

Last updated by author(s): Apr 9, 2022

Reporting Summary

Nature Research wishes to improve the reproducibility of the work that we publish. This form provides structure for consistency and transparency in reporting. For further information on Nature Research policies, see our Editorial Policies and the Editorial Policy Checklist.

Statistics

For all statistical analyses, confirm that the following items are present in the figure legend, table legend, main text, or Methods section.

n/a	Confirmed	
☐	☒	The exact sample size (*n*) for each experimental group/condition, given as a discrete number and unit of measurement
☐	☒	A statement on whether measurements were taken from distinct samples or whether the same sample was measured repeatedly
☐	☒	The statistical test(s) used AND whether they are one- or two-sided<br>*Only common tests should be described solely by name; describe more complex techniques in the Methods section.*
☐	☒	A description of all covariates tested
☒	☐	A description of any assumptions or corrections, such as tests of normality and adjustment for multiple comparisons
☐	☒	A full description of the statistical parameters including central tendency (e.g. means) or other basic estimates (e.g. regression coefficient) AND variation (e.g. standard deviation) or associated estimates of uncertainty (e.g. confidence intervals)
☐	☒	For null hypothesis testing, the test statistic (e.g. *F*, *t*, *r*) with confidence intervals, effect sizes, degrees of freedom and *P* value noted<br>*Give P values as exact values whenever suitable.*
☒	☐	For Bayesian analysis, information on the choice of priors and Markov chain Monte Carlo settings
☒	☐	For hierarchical and complex designs, identification of the appropriate level for tests and full reporting of outcomes
☒	☐	Estimates of effect sizes (e.g. Cohen's *d*, Pearson's *r*), indicating how they were calculated

Our web collection on statistics for biologists contains articles on many of the points above.

Software and code

Policy information about availability of computer code

Data collection	Microscopy data were collected using Fusion 2.0.<br>TIRF imaging data were collected using cellSens Dimension 1.17.<br>Seahorse data were acquired using a Seahorse Wave software.<br>Mechanical model was based on the finite element method using Abaqus 6.14.
Data analysis	Data were analyzed with Microsoft Excel 2019 and Prism 6.<br>Hessian SIM imaging data were analyzed using a custom MATLAB algorithm.<br>Images were processed (crop, overlay, colors) with Image J 1.52p.<br>FIB-SEM reconstruction was performed on Amira software.<br>Details are described in the Methods section.

For manuscripts utilizing custom algorithms or software that are central to the research but not yet described in published literature, software must be made available to editors and reviewers. We strongly encourage code deposition in a community repository (e.g. GitHub). See the Nature Research guidelines for submitting code & software for further information.

Data

Policy information about availability of data

All manuscripts must include a data availability statement. This statement should provide the following information, where applicable:

- Accession codes, unique identifiers, or web links for publicly available datasets
- A list of figures that have associated raw data
- A description of any restrictions on data availability

????
The data that support the findings of this study are available within the paper and its Supplementary Information files.

Field-specific reporting

Please select the one below that is the best fit for your research. If you are not sure, read the appropriate sections before making your selection.

☒ Life sciences ☐ Behavioural & social sciences ☐ Ecological, evolutionary & environmental sciences

For a reference copy of the document with all sections, see nature.com/documents/nr-reporting-summary-flat.pdf

Life sciences study design

All studies must disclose on these points even when the disclosure is negative.

Sample size	No statistical methods were used to predetermine sample size. Sample sizes were chosen based on commonly adopted standards, resulting in statistically meaningful comparisons. Multiple independent experiments were executed by using several sample replicates as detailed in figure legends to ensure these sample sizes are sufficient.
Data exclusions	No data was excluded.
Replication	All experiments were executed under standard and clearly defined conditions, and were replicated successfully by at least one researcher for at least three times. All attempts at replication were successful.
Randomization	Cells or microscopy fields were randomly allocated into experimental groups.
Blinding	Imaging data were captured blindly by selecting cells or microscopy fields.

Reporting for specific materials, systems and methods

We require information from authors about some types of materials, experimental systems and methods used in many studies. Here, indicate whether each material, system or method listed is relevant to your study. If you are not sure if a list item applies to your research, read the appropriate section before selecting a response.

Materials & experimental systems

n/a	Involved in the study	
☐	☒	Antibodies
☐	☒	Eukaryotic cell lines
☒	☐	Palaeontology and archaeology
☒	☐	Animals and other organisms
☒	☐	Human research participants
☒	☐	Clinical data
☒	☐	Dual use research of concern

Methods

n/a	Involved in the study	
☒	☐	ChIP-seq
☐	☒	Flow cytometry
☒	☐	MRI-based neuroimaging

Antibodies

Antibodies used	The following antibodies were used in this study: rabbit anti-Myo19 (HPA059715, 1:1000 for westernblotting) from Sigma-Aldrich; rabbit anti-Mic60 (A2751, 1:3000 for wesernblotting) , rabbit anti-Tom20 (A19403, 1:4000 for westernblotting, 1:400 for immunofluorescence staining), mouse anti-MBP(AE016,1:2000 for westernblotting), rabbit anti-Miro1 (A5838, 1:500 for westernblotting),rabbit anti-Metaxin2 (A7958, 1:1000 for westernblotting)and mouse anti-Sam50 (A3401, 1:3000 for westernblotting, 1:400 for PLA assay) from ABclonal; mouse anti-Mic60 (sc-390707, 1:200 for immunofluorescence staining) from Santa Cruz Biotechnology, mouse anti-Flag(M185, 1:3000 for westernblotting, 1:200 for IP) and mouse anti-GFP (MO48-3, 1:200 for immunofluorescence staining) from MBL; rabbit anti-Miro2 (11237-1-AP, 1:1000 for westernblotting), from Proteintech, anti-mouse (sc-516102, 1:4000) and anti-rabbit (sc-2004, 1:4000) horseradish peroxidase (HRP)-conjugated secondary antibodies from Santa Cruz Biotechnology.

Validation | All antibodies used have been obtained from commercial vendors. Validation information can be found from suppliers websites.

Eukaryotic cell lines

Policy information about cell lines

Cell line source(s)	MDA-MB-231 cells and Human embryonic kidney 293T (HEK 293T) cells were from ATCC.
Authentication	MDA-MB-231 cells and Human embryonic kidney 293T (HEK 293T) cells were obtained pre-authenticated directly from ATCC.
Mycoplasma contamination	Original cells are not mycoplasma contaminated.
Commonly misidentified lines (See ICLAC register)	No cell lines used in this study were found in ICLAC register.

Flow Cytometry

Plots

Confirm that:

- [x] The axis labels state the marker and fluorochrome used (e.g. CD4-FITC).
- [x] The axis scales are clearly visible. Include numbers along axes only for bottom left plot of group (a 'group' is an analysis of identical markers).
- [ ] All plots are contour plots with outliers or pseudocolor plots.
- [ ] A numerical value for number of cells or percentage (with statistics) is provided.

Methodology

Sample preparation	Cells were trypsinized and the suspension cells were stained with 100 nM TMRM for 20 minutes. For positive control, 5 µM FCCP was added and stained simultaneously for 10 minutes. Then, cells were washed using pre-warmed DPBS.
Instrument	BD FACSCanto II.
Software	FlowJo_V10.
Cell population abundance	N/A
Gating strategy	Cells without TMRM staining was used to determine gating.

- [ ] Tick this box to confirm that a figure exemplifying the gating strategy is provided in the Supplementary Information.
